# Acute systemic loss of Mad2 leads to intestinal atrophy in adult mice

**DOI:** 10.1038/s41598-020-80169-5

**Published:** 2021-01-08

**Authors:** Klaske M. Schukken, Yinan Zhu, Petra L. Bakker, Mirjam H. Koster, Liesbeth Harkema, Sameh A. Youssef, Alain de Bruin, Floris Foijer

**Affiliations:** 1 European Research Institute for the Biology of Ageing (ERIBA), University of Groningen, University Medical Center Groningen, 9713 AV Groningen, The Netherlands; 2 Dutch Molecular Pathology Center, Department of Biomolecular Health Sciences, Faculty of Veterinary Medicine, Utrecht University, 3584 CL Utrecht, The Netherlands; 3 Department of Pediatrics, University of Groningen, University Medical Center Groningen, 9713 AV Groningen, The Netherlands; 4 Present Address: Cold Spring Harbor Laboratories, Cold Spring Harbor, USA; 5 Present Address: GD Animal Health, 7418EZ Deventer, The Netherlands; 6 Present Address: Janssen Research and Development, 2340 Beerse, Belgium

**Keywords:** Cancer models, Disease model, Stem cells

## Abstract

Chromosomal instability (CIN) is a hallmark of cancer, leading to aneuploid cells. To study the role that CIN plays in tumor evolution, several mouse models have been engineered over the last 2 decades. These models have unequivocally shown that systemic high-grade CIN is embryonic lethal. We and others have previously shown that embryonic lethality can be circumvented by provoking CIN in a tissue-specific fashion. In this study, we provoke systemic high-grade CIN in adult mice as an alternative to circumvent embryonic lethality. For this, we disrupt the spindle assembly checkpoint (SAC) by alleviating Mad2 or truncating Mps1, both essential genes for SAC functioning, with or without p53 inactivation. We find that disruption of the SAC leads to rapid villous atrophy, atypia and apoptosis of the epithelia of the jejunum and ileum, substantial weight loss, and death within 2–3 weeks after the start of the CIN insult. Despite this severe intestinal phenotype, most other tissues are unaffected, except for minor abnormalities in spleen, presumably due to the lower proliferation rate in these tissues. We conclude that high-grade CIN in vivo in adult mice is most toxic to the high cell turnover intestinal epithelia.

## Introduction

Chromosomal instability (CIN) is a process that leads to cells with an abnormal DNA content, a state also known as aneuploidy. The Spindle Assembly Checkpoint (SAC) helps to prevent CIN by arresting cells in metaphase until all chromosomes are properly attached to the spindle network. Inhibiting any protein involved in the SAC leads to high levels of CIN^1–3^, including the kinase Mps1, which regulates checkpoint protein binding to kinetochores and Mad2, which inhibits the anaphase promoting complex (APC/c) when bound to kinetochores^4–10^.

CIN is a hallmark of cancer cells^1,11–13^, and while various mechanisms can lead to chromosome miss-segregation^5,14–19^, SAC alleviation is commonly used as a tool to provoke CIN in model systems. While SAC genes are rarely mutated^20^, SAC function has been found to be impaired in various human cancers^20,21^. Several mouse models have been engineered to study the effect of CIN on cancer initiation and progression^1–3^. These models have unequivocally shown that systemic high-grade CIN is incompatible with embryonic development and further that low-grade CIN predisposes to cancer, most efficiently when combined with other cancer predispositions such as p53 loss or APC mutation^1–3,22^. To circumvent CIN-induced embryonic lethality^5,23^, conditional mouse models were engineered in which CIN can be provoked in a tissue-specific fashion to study the consequences of chromosome mis-segregation in individual tissues in vivo^1,5^. One intriguing finding from these models is that some tissues, but not all, tolerate high levels of CIN. For instance, inducing CIN by inactivating Mad2 is incompatible with embryonic development^1^, often lethal to cultured cells^24^, and toxic to hair follicle stem cells^25^, but tolerated by basal epidermal cells^25^, T-cells^26^, and hepatocytes in vivo^26^. Furthermore, systemic CIN provoked in adult mice drives tumorigenesis in a dose-dependent fashion with medium CIN rates being most efficient^22^. Here, we study the effects of acute high-grade CIN in adult mice provoked by complete SAC alleviation and find that this leads to rapid regression of intestinal epithelia followed by significant weight loss and ultimately death.

## Material and methods

### Animal experiments and genotyping

All animal experiments were performed in accordance with Dutch law and approved by the University of Groningen Medical Center Committees on Animal Care. Animal experiments were carried out and animal experiment protocols were described according to the ARRIVE 2.0 guidelines^27^. The conditional *Mad2*^*f/f*^ and *Mps1*^*f/f*^ animal models were previously described^10,25,26^. Mice used in this study had a mixed 129/Sv and C57BL-6 background. *Trp53*^*f/f*^ (*p53*^*f/f*^) conditional knockout mice were a gift from Jos Jonkers^28^. All mice used were between 9 and 10 weeks old. *Mad2*^*f/f*^ or *Mps1*^*f/f*^ mice were bred with ubiquitous Cre‐ERT2 mice^29^ and *p53*^*f/f*^ mice to obtain the various cohorts of mice described in this study. Mice and tissues were genotyped for *Mad2*^*flox*^^26^, *Mps1*^*flox*^^10^, *p53*^*flox*^^26^, *Cre-ERT2*^29^ and *Mad2*^*∆*^^26^ as described previously.

### Tamoxifen induction

To find the optimal method of tamoxifen administration, three different administration methods were compared in a small cohort of mice, which included tamoxifen provided in food pellets (Envigo, 250 mg/kg), tamoxifen in peanut oil injected intraperitoneally or by oral gavage. As mice put on a tamoxifen-containing diet had lower food intake than mice on a normal diet, and IP injections come with a risk of intestinal punctures, we used oral gavage for the experiment cohort. For IP injections and oral gavage, we administrated 0.13 mg tamoxifen (Sigma) per gram of mouse in peanut oil (Sigma).

### Histological analysis

Animals were euthanized, organs removed and washed with PBS. Tissues were placed in a cassette, and fixed in 10% neutral buffered formalin for 1–2 days, then moved into 70% ethanol. Histology slides were prepared and standard H&E staining was performed at the Department of Pathology at Utrecht University. For cleaved Caspase 3 stainings, paraffin-preserved tissue sections were stained with a rabbit anti human/mouse cleaved Caspase-3-active primary antibody (R&D systems, AF835) followed by incubation with a goat anti-rabbit/biotin secondary antibody (Vector laboratories Inc., BA-1000); avidin–biotin-complex (Vector Laboratories Inc., PK-4000). Slides were next scanned on a slide scanner (Leica) and analyzed as described below.

### Statistical analysis and plots

Intestinal villi length, crypt length, red blood cell diameter and number of Howell‐Jolly bodies present in blood samples were compared with a two‐sided t test. A Wilcox rank sum test was used for categorical data, and data with values near zero, as indicated in Tables 1 and 2, including the analysis of mitotic abnormalities. A two‐sided t test was used to evaluate the difference in mouse weight change (relative to day 0) per group, comparing experimental to control mice. A log‐rank (Mantel–Cox) test was used to compare survival curves. Survival curves and weight loss graph were plotted in GraphPad PRISM.

**Table 1:** Histological data for jejunum/ileum per genotype.

Genotype	Mouse ID	Jejunum/ileum								Apoptotic cells in 5 HPF
		Crypt depth (um)	Villus length (um)	Villus atrophy	Villus epithelium degeneration	Villus epithelium atypia	Crypt epithelial degeneration	Crypt epithelial atypia	Crypts	
Mad2^f/f^ p53^f/f^	118	100	350	1	1	1.5	2	2.5	3	102
Mad2^f/f^ p53^f/f^	121	115	275	2	1	2.5	2	2.5	3	127
Mad2^f/f^ p53^f/f^	123	85	355	1	1	1.5	2	2	3	158
Mad2^f/f^ p53^f/f^	135	90	280	2	2	3	2.5	3	3	96
Mad2^f/f^	127	90	330	1	1.5	2.5	2	3	2	54
Mad2^f/f^	129	80	220	2.5	0	3	3	2.5	3	36
Mad2^f/f^	131	100	300	1	1	1	2	2	2	59
Mad2^f/f^	143	80	200	2.5	1	3	2.5	3	3	38
Control	138	105	520	0	0	0	0	0	0	0
Control	139	85	300	1	0	0	0	0	0	0
Control	140	105	535	0	0	0	0	0	0	0
Control	125	80	500	0	0	0	0	0	0	0
		t-test		Wilcox sum rank test						
Mad2^f/f^ p53^f/f^ vs. control		0.70	0.046	0.047	0.018	0.020	0.018	0.020	0.013	0.021
Mad2^f/f^ vs. control		0.47	0.019	0.047	0.067	0.020	0.020	0.020	0.019	0.021

Villus atrophy^38^: 0 = absent, 1 = mild: ~ 75% of normal length; 2 = moderate: ~ 50% of normal length; 3 = Marked: < 25% of normal length.

Villus epithelium degeneration: 0 = absent; 1 = mild: epithelial attenuation, loss of brush border; 2 = moderate: clear epithelial degeneration; 3 = severe: loss of epithelial cells.

Villus epithelium atypia: 0 = absent, 1 = mild, 2 = moderate, 3 = severe.

Crypt epithelial degeneration: 0 = absent; 1 = mild: epithelial attenuation; 2 = moderate: clear epithelial degeneration; 3 = severe: sloughing and loss of epithelial cells.

Crypt epithelial atypia: 0 = absent, 1 = mild, 2 = moderate, 3 = severe.

Crypts^38^: 0 = normal, 1 = 10% ; 2 = 10–50%; 3 =  > 50% of the crypts contain apoptotic cells/debris.

Note that numbers in the tables that describe these tissue states are sometimes non-integer numbers. In this case the condition of the tissue was in between both states that the above described integers refer to.

**Table 2:** Histological data of organs per genotype: spleen, blood. lung liver and kidneys.

		Spleen			Other tissues			Blood			
Genotype	Mouse ID	Spleen atypical	Spleen EMH	Spleen follicles	Lung	Liver	Kidney	Rbc diameter (μm)	Polychromatophilia	Size of polychromatic cells (μm)	Howell–Jolly bodies
Mad2^f/f^ p53^f/f^	118	3	1	nsl	nsl	nsl	nsl	6–6.5	0		4/HPF
Mad2^f/f^ p53^f/f^	121	3	1	nsl	nsl	nsl	nsl	6–6.5	1/HPF		4/HPF
Mad2^f/f^ p53^f/f^	123	2.5	1.5	nsl	nsl	nsl	nsl	5.5–6	0		1/HPF
Mad2^f/f^ p53^f/f^	135	2	1	nsl	nsl	nsl	nsl	6	0		3/HPF
Mad2^f/f^	127	0.5	1.5	nsl	nsl	nsl	nsl	5.5–6	0		3/HPF
Mad2^f/f^	129	0	1.5	nsl	nsl	nsl	nsl	6–6.5	0		2/HPF
Mad2^f/f^	131	0.5	1	nsl	nsl	nsl	nsl	5.5–6	0		1/HPF
Mad2^f/f^	143	0	0	nsl	nsl	nsl	nsl	6–6.5	0		4/HPF
Control	138	0	2	nsl	nsl	nsl	nsl	6–6.5	6/HPF	7	1/HPF
Control	139	0	2	nsl	nsl	nsl	nsl	6.5	20/HPF	6.5–7	6/HPF
Control	140	0	2	nsl	nsl	nsl	nsl	5.5–6.5	25/HPF	6.5–7	2/HPF
Control	125	0	2	nsl	nsl	nsl	nsl	6	13/HPF	6.5–7	1/HPF
		Wilcox sum rank test						t test			t test
Mad2^f/f^ p53^f/f^ vs*.* control		0.020	0.018					ns			0.73
Mad2^f/f^ vs*.* control		0.018	0.020					ns			1

*nsl* no significant lesions, *HPF* high power fields at 400×. The area visible under a high magnification microscope at 400×.

Spleen atypical: atypical cells within the red pulp: scores: 0 = absent; 0.5 = rarely single atypical cell present; 1 = 1–5 per HPF; 2 = 5–10 per HPF; 3 = 10–20 per HPF.

Spleen EMH (extramedullary hematopoiesis within the red pulp): scores: 0 = absent; 1 = mild; 2 = moderate; 3 = marked.

## Results

CIN driven by systemic Mad2 inactivation is incompatible with mouse embryonic development^23^. However, the impact of acute systemic Mad2 loss or acute Mps1 truncation in adult mice, circumventing embryonic defects, has not been tested. For this purpose, we crossed *Mad2*^*f/f*^^25,26^ and *Mps1*^*f/f*^^10^ conditional mice with mice expressing a ubiquitous tamoxifen-inducible Cre recombinase (*Cre-ERT2)*^29^. In these mice, tamoxifen treatment yields rapid and systemic Mad2 loss or acute Mps1 truncation. Since p53 loss can partly rescue cell death induced by Mad2 loss in vivo and in cultured ES cells^6^, we also crossed the *Mps1*^*f/f*^*; Cre-ERT2* and *Mad2*^*f/f*^*; Cre-ERT2* strains into a *p53* conditional knockout background^28^.

Next, we compared protocols for tamoxifen administration: intraperitoneal (IP) injections, oral gavage and tamoxifen administration through food pellets and compared the effects of the *Mps1* and *Mad2* alleles as CIN drivers, with and without *p53* conditional alleles (Supplementary Fig. S1A,B). We did not observe significant differences between the administration route of tamoxifen, the CIN-driving alleles, nor p53 status: most mice had to be sacrificed because of excessive weight loss within 2 weeks or 3 weeks in case Cre-ERT2 was activated through tamoxifen supplied in food pellets (Supplementary Fig. S1A,B). As IP injections come with a risk of intestinal punctures and mouse food intake was reduced for tamoxifen-containing food pellets, also in control mice, in follow up experiments, tamoxifen was administrated by oral gavage. Furthermore, as we did not observe any differences in the phenotypes of mice with *Mad2*^*f/f*^ and *Mps1*^*f/f*^ alleles, to minimize mouse numbers, we only pursued *Mad2;* (*p53*) conditional mice.

**Figure 1:**
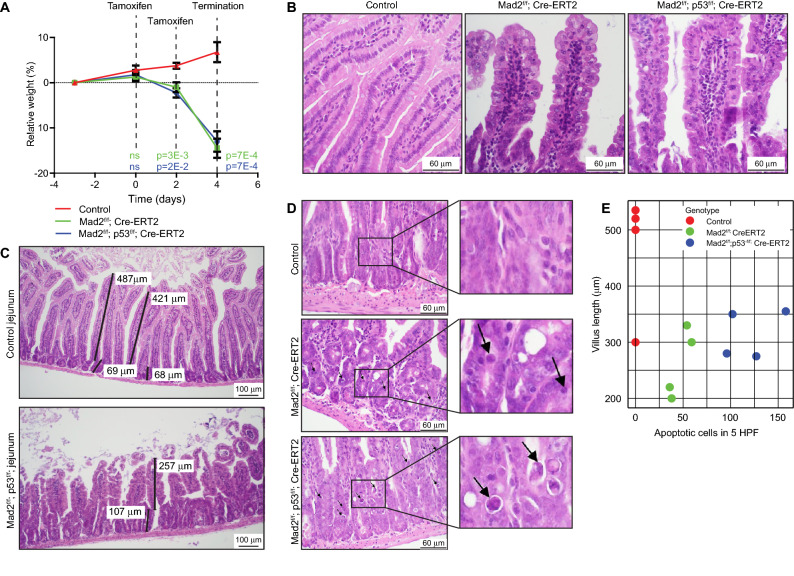
Inactivation of Mad2 knockout leads to rapid atrophy in jejunum/ileum. (**A**) Relative weight compared to starting weight over time. Plotted average weight with standard error of the mean (SEM), n = 4 per condition. Two-sided t test was used to test difference between each genetic group and the control, P value per timepoint and per genotype displayed. (**B**) Representative images of jejunum of control and Mad2; p53 compound knockout mice. H&E staining, scale bar is 60 μm. (**C**) Representative images of villi and crypts of the jejunum showing normal jejunum (top panel) and *Mad2*^*f/f*^*; p53*^*f/f*^*; Cre-ERT2* jejunum with atrophied villi and atypical cells (bottom panel). H&E staining, scale bar 100 μm. Lines and numbers represent the crypt and villus length, and are quantified for multiple images in (**E**). (**D**) Representative images of villi and crypts of the jejunum per genotype. H&E staining, scale bar is 60 μm. Black arrows point to apoptotic cells. (**E**) Scatter plot of villus length and apoptotic cells per high power field (HPF) image per genotype.

To understand the cause of the excessive weight loss, we next setup a new cohort of 4 *Mad2*^*f/f*^* Cre-ERT2* and 4 *Mad2*^*f/f*^;*p53*^*f/f*^*;Cre-ERT2* mice. As controls, we included two Cre‐ERT2 mice that received control-vehicle instead of tamoxifen, and two Cre‐ERT2 negative mice that received tamoxifen. While control mice gained an average of 7% of their body weight; *Mad2*^*f/f*^; *Cre‐ERT2* and *Mad2*^*f/f*^*;p53*^*f/f*^;*Cre‐ERT2* mice lost 14% and 13% of their weight, respectively within 4 days after treatment had started (Fig. 1A).

Next, mice were euthanized 4 days after tamoxifen treatment had started and tissues harvested for analysis. We inspected the jejunum, spleen, lung, liver, kidneys, and a blood smear for each mouse and found no overt abnormalities in most tissues, except for jejunum/ileum and spleen (Tables 1, 2). To test whether this phenotype indeed correlated to loss of Mad2 in the affected tissues, we next assessed by genomic PCR whether tamoxifen treatment had resulted in conversion of the *Mad2* conditional allele (*Mad2*^*f*^) into a *Mad2* deletion allele (*Mad2*^*∆*^) and found that in all samples, except for those isolated from mice that were not exposed to tamoxifen or did not carry a Cre-ERT2 conditional allele, the *Mad2* deletion product was readily detectable, indicating that the *Mad2* coding sequence was removed in a large fraction of the tissue (Supplementary Fig. S1C). Note that Mad2 was not completely lost, i.e. we could still detect the *Mad2*^*flox*^ allele (Supplementary Fig. S1C, upper band), indicating that Mad2 loss was mosaic throughout the tissue and thus that mosaic Mad2 loss was sufficient to trigger the observed phenotypes.

**Figure 2:**
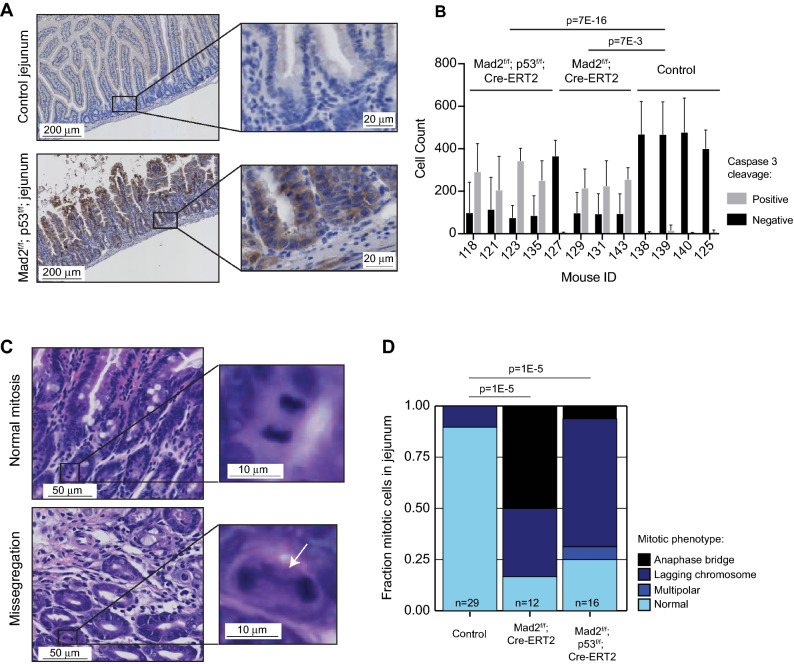
Acute Mad2 loss leads to apoptosis and mitotic abnormalities in intestine. (**A**) Representative images of control and *Mad2*^*f/f*^*; p53*^*f/f*^*; Cre-ERT2* jejunum/ileum immuno-stained for cleaved Caspase3. Insets show zoom-in of intestinal crypts. (**B**) Quantification of fraction of Caspase 3-cleaved cells in intestine for each assessed mouse. Minimum of six representative images per mouse, and minimum of 100 cells quantified per image. P value is shown for a t test between the three genotypes. Error bars indicate the SEM. (**C**) Representative images of a normal mitosis (upper panel) and abnormal mitosis (bottom panel) in the intestinal crypt. H&E staining of mouse jejunum. (**D**) Quantification of the observed mitotic abnormalities per genotype assessed from tissue samples. Chi-squared test comparing cumulative mitotic abnormalities to normal mitosis per condition. “n” refers to the total number of mitotic events quantified per genotype.

When evaluating the jejunum/ileum, we noticed several atypia of the mucosal cells of the villi, including abnormal columnar shape of villi (Fig. 1B, compare shape of villi top and bottom panel Fig. 1C). We also observed a significant increase in cells with enlarged nuclei (karyomegaly) in the knockout mice, with both *Mad2*^*f/f*^*; p53*^*f/f*^*; Cre-ERT2* and *Mad2*^*f/f*^*; Cre-ERT2* mice displaying more enlarged nuclei than control mice (Supplementary Fig. S2A,B), in agreement with the expected chromosomal instability imposed by Mad2 loss. Most strikingly, the knockout mice displayed a significant decrease in villus length in the jejunum (Fig. 1C), which coincided with a significant increase in atypical cells with less condensed nuclei that were likely apoptotic (Fig. 1D, Table 1). While survival or weight loss kinetics between p53-proficient and -deficient mice were comparable, the jejunum/ileum of *Mad2*^*f/f*^;*p53*^*f/f*^* Cre-ERT2* mice displayed a higher number of such abnormally shaped cells with less condensed chromatin than *Mad2*^*f/f*^ mice, suggesting an increased frequency of mitotic catastrophe in *Mad2*^*f/f*^;*p53*^*f/f*^;*Cre-ERT2* mice (Fig. 1D,E), a p53-independent type of apoptosis^30^. To confirm and quantify apoptosis in the intestinal samples, we performed a cleaved Caspase-3 staining and found that both *Mad2*^*f/f*^*; Cre-ERT2* as well as *Mad2*^*f/f*^*; p53*^*f/f*^*; Cre-ERT2* genotypes displayed a dramatic increase of apoptotic cells with up to ~ two-third of the cells staining positive for cleaved Caspase 3, while control jejunum barely harbored any apoptotic cells (Fig. 2A,B, Supplementary Fig. S2A,B). Notably, all (19 out of 19) atypical cells (enlarged, with less condensed nuclei) stained positive for cleaved Caspase 3 (Supplementary Fig. S2C). To link this phenotype to chromosome missegregation, we next quantified abnormal mitotic figures in the tissues (Fig. 2C,D), which revealed that up to 75% of the mitotic cells in *Mad2*^*f/f*^*; Cre-ERT2* and *Mad2*^*f/f*^*; p53*^*f/f*^*; Cre-ERT2* jejunum/ileum displayed abnormalities (anaphase bridges, lagging chromosomes or multipolar divisions), indicating that the intestinal atrophy and apoptotic cells resulted from chromosomal abnormalities imposed by Mad2 loss. We conclude that in these mice Mad2 loss imposed a CIN phenotype that lead to apoptotic cells and thus atrophy in the jejunum/ileum. The observed apoptosis and atrophy most likely interfered with the uptake of nutrients, causing extreme weight loss ultimately leading to death of the mice. This phenotype is consistent with the high turnover of intestinal cells: intestinal villi proliferate rapidly and are renewed every 3–5 days^31^.

**Figure 3:**
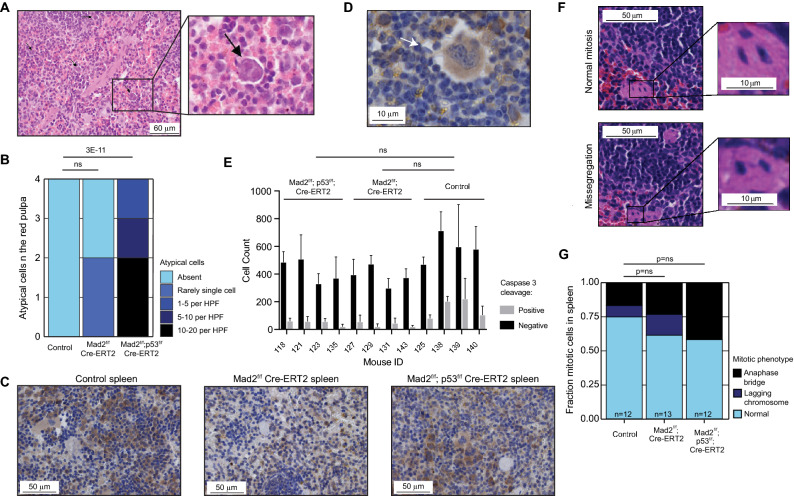
Acute Mad2 loss leads to a modest phenotype in spleen within the first 4 days (**A**) *Mad2*^*f/f*^*; p53*^*f/f*^; Cre-ERT2 spleen showing cellular atypia in the red pulp, scale bar 20 μm, HE staining, magnification ×400. (**B**) Quantification of spleen abnormalities between genotypes, n = 4 per genotype. P values refer to Wilcox rank‐sum test. (**C**) Representative images of mouse spleen immuno-stained for cleaved Caspase3. (**D**) Zoom-in of an atypical cell positive for cleaved Caspase 3. (**E**) Quantification of fraction of Caspase 3-cleaved cells in spleen for each assessed mouse. Minimum of six representative images per mouse, and minimum of 100 cells quantified per image. P value is shown for a t test between the three genotypes, P < 0.01 was considered significant. Error bars indicate the SEM. (**F**) Representative images of a normal mitosis (upper panel) and abnormal mitosis (bottom panel) in the intestinal crypt. H&E staining of mouse jejunum. (**G**) Quantification of the observed mitotic abnormalities per genotype assessed from tissue samples. Chi-squared test comparing cumulative mitotic abnormalities to normal mitosis per condition. “n” refers to the total number of mitotic events quantified per genotype.

In addition to the abnormalities observed in the jejunum/ileum, we also observed large, atypical cells with less condensed chromatin within the red pulp of the spleen in *Mad2*^*f/f*^;*p53*^*f/f*^;*Cre-ERT2* mice (Fig. 3A,B, Table 2), and to a lesser extent in two of the four *Mad2*^*f/f*^;*Cre-ERT2* mice. These atypical cells showed a high nuclear/cytoplasm ratio with nuclear atypia, again in line with a CIN phenotype. While all of these atypical cells (13 out of 13 quantified cells) were positive for cleaved Caspase 3, overall the apoptotic rates were not increased in *Mad2*^*f/f*^*; Cre-ERT2 and Mad2*^*f/f*^*; p53*^*f/f*^*; Cre-ERT2* splenic samples (Fig. 3C–E). Although the atypical cells were unlikely to be the cause of the extreme weight loss and death, these changes might thus represent mild hematopoietic dysplasia related to the first effects of Mad2 (and p53) loss in the hematopoietic system. Next, we quantified mitotic abnormalities in spleen (Fig. 3F). Even though there was a modest trend of increasing mitotic abnormalities in *Mad2*^*f/f*^*; Cre-ERT2 and Mad2*^*f/f*^*; p53*^*f/f*^*; Cre-ERT2*, respectively, this was not significant (Fig. 3G), in agreement with the overall milder phenotype in spleen as compared to jejunum/ileum. Finally, we did not find abnormalities in the blood smears except for a small decrease in polychromatic cells, indicative of decreased red blood cell production^32^ (Table 2), suggesting that the lymphoid cells in peripheral blood were not affected within the first 4 days of tamoxifen treatment.

We conclude that acute systemic loss of Mad2 with or without p53 inactivation causes rapid atrophy of intestinal epithelia yielding reduced nutrient uptake, ultimately leading to death. While this phenotype is intriguing and suggests that Mad2 loss is particularly toxic to the stem cells residing in the intestinal crypts, similar to what was observed previously for hair follicle stem cells^25^, the severity of the phenotype also precluded assessment of Mad2 loss in other adult tissues.

## Discussion

Systemic inactivation of the SAC, and the resulting high levels of CIN, lead to early embryonic death^1–3^. However, to our knowledge, the consequences of complete systemic SAC alleviation in adult mice have not been reported so far. We have previously shown that tissue-specific inactivation of the SAC can circumvent embryonic lethality associated with Mad2 loss^25,26^, and found that different tissues cope differently with SAC loss. For instance, inactivation in the epidermis revealed that SAC alleviation is not tolerated by hair follicle stem cells, but remarkably well-tolerated by the basal cells of the epidermis^25^. In this study, we find that systemic inactivation of the SAC leads to rapid death of adult mice coinciding with rapid weight loss. The rapid weight loss following Mad2 loss coincides with mitotic abnormalities and increased apoptosis of proliferating cells in the intestinal crypts of the jejunum/ileum, leading to a severe atrophy of intestinal epithelia.

While we observed a significant phenotype in jejunum/ileum, we did not find any noticeable effects of SAC loss in lung, liver, and kidneys within the 4-day timeframe. We did observe a weak phenotype in the hematopoietic system, mostly in spleen. A possible explanation for this is the proliferation rate within these tissues, as both intestinal crypt cells and hematopoietic stem cells produce rapidly proliferating cells^31,33,34^. Notably, while loss of p53, or p53 mutation enhances CIN tolerance in MEFs^6^, we did not observe striking differences between the phenotypes of *Mad2*^*f/f−*^*; Cre-ERT2* and *Mad2*^*f/f*^*; p53*^*3f/f*^;* Cre-ERT2* adult mice, suggesting that in jejunum/ileum p53 loss is not sufficient to rescue apoptotic cell death in a high-grade CIN background*.* As Mad2 loss is particularly toxic to hair follicle stem cells, but not to the basal cells in mouse epidermis^25^, it is tempting to extrapolate these findings to intestine, where the stem cells reside in the crypts and their differentiated progeny moves up in the villi^35^. Indeed, the crypts of jejunum/ileum of the *Mad2*^*f/ −*^*; Cre-ERT2* and *Mad2*^*f/f*^*; p53*^*3f/f*^*; Cre-ERT2* mice show many apoptotic cells (zoomed-in sections Fig. 2A), indicating that these cells indeed cope very poorly with CIN. This is also true for the transit-amplifying cells higher up in the villi and their differentiated progeny. However, as all differentiated cells in the villi stem from the stem cells in the crypt and the complete villi are renewed every 4–5 days, novel intravital imaging models will be required to assess differences in cell fate of aneuploid stem cells, transit amplifying cells and their differentiated progeny. Overall, our data suggest that acute Mad2 alleviation is particularly toxic to intestine as this tissue completely renews every 3–5 days^31^ for which it completely relies on the stem cells in the crypts, which apparently cope very poorly with CIN.

Since CIN is a hallmark of cancer cells, and three out of four tumors are aneuploid^11,12^, it is of the utmost importance to know to what extent individual tissues tolerate CIN in vivo, as this will contribute to our understanding of cancer progression per tissue type. Furthermore, many commonly-used cancer therapeutics, including taxanes and vinca-alkaloids trigger a CIN phenotype in cultured cells^36^, but it is not trivial to prove that their therapeutic impact in vivo also results from missegregation events and not other mechanisms. Interestingly, the side-effects of these therapies, which include intestinal atrophy and anemia^37^, are very similar to what we have observed in our here-described mouse model. Therefore, our data provides further evidence that microtubule poisons also trigger a CIN phenotype in vivo, thus further improving our understanding of how these therapies work in vivo.

## Supplementary Information

Supplementary Information
