# Supplementary material for: Acute systemic loss of Mad2 leads to intestinal atrophy in adult mice
Source: Sci Rep. 2021 Jan 8;11:68. doi: 10.1038/s41598-020-80169-5 (PMC7794249; doi:10.1038/s41598-020-80169-5)
Supplement: Supplementary file 1 — Supplementary Information [file 41598_2020_80169_MOESM1_ESM.pdf]

Supplementary data: ***Acute systemic loss of Mad2 leads to intestinal atrophy in adult mice***

Klaske M. Schukken[1,a], Yinan Zhu[1], Petra L. Bakker[1], Mirjam H. Koster[3], Liesbeth Harkema[2,b], Sameh A. Youssef[2,3,c], Alain de Bruin[2,3], and Floris Foijer[1,*]

[1]European Research Institute for the Biology of Ageing (ERIBA), University of Groningen, University Medical Center Groningen, 9713 AV, Groningen, the Netherlands.
[2]Dutch Molecular Pathology Center, Department of Biomolecular Health Sciences, Utrecht University, Faculty of Veterinary Medicine, 3584 CL, Utrecht, the Netherlands.
[3]Department of Pediatrics, University of Groningen, University Medical Center Groningen, 9713 AV, Groningen, the Netherlands
[a] current address: Cold Spring Harbor Laboratories, Cold Spring Harbor, USA.
[b] current address: GD Animal Health, 7418EZ Deventer, the Netherlands
[c] current address: Janssen Research and Development, 2340 Beerse, Belgium

[*]To whom correspondence should be addressed: f.foijer@umcg.nl

Contents:

Supplementary Figure 1: Kaplan-Meier curves per genotype for several tamoxifen administration routes and genomic PCRs to show *Mad2* loss in intestine and spleen. (**A, B**) Kaplan-Meier curves for (A) *Mad2$^{f/f}$; Cre-ERT2 and Mad2$^{f/f}$; p53$^{f/f}$; Cre-ERT2*, or (**B**) *Mps1$^{f/f}$; Cre-ERT2* and *Mps1$^{f/f}$; p53$^{f/f}$; Cre-ERT2* mice for various routes of tamoxifen administration. Number of mice per genotype/treatment type is listed. (**C**) Genomic PCRs for *Mad2^{flox}* and *Mad2$^{\Delta}$* on DNA isolated from jejunum/ileum (top panel) or spleen (bottom panel).

Supplementary Figure 2: Karyomegaly in Mad2-deficient intestine. (**A**) Representative images of cells with enlarged nuclei (karyomegalic cells) in mouse jejunum immuno-stained for active Caspase3. Arrows point to karyomegalic cells. (**B**) Mean frequency of karyomegaly per HPF, minimum of 6 HPF per mouse, 4 mice per genotype. p-values determined with two-sided t-test. (**C**) Representative image of atypical cell in mouse jejunum immuno-stained for active Caspase3. White arrows point to atypical cells.

Supplementary Figure 3: Raw uncropped microscopy images and genomic PCR gel used for main and supplementary figures (multiple pages, one raw image per page with reference to which figure this data contributed to).

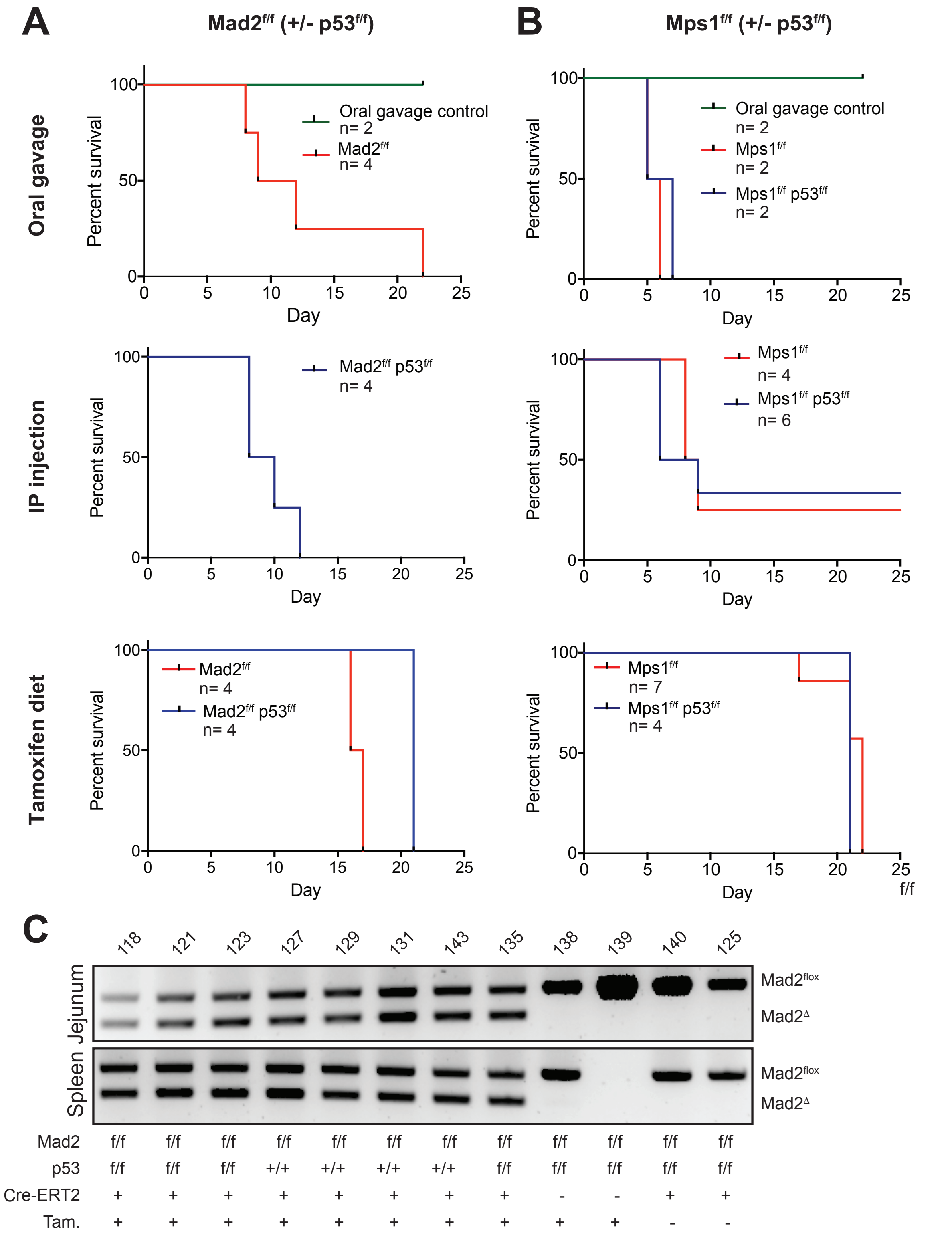

Schukken *et al*, Sup. Figure 1

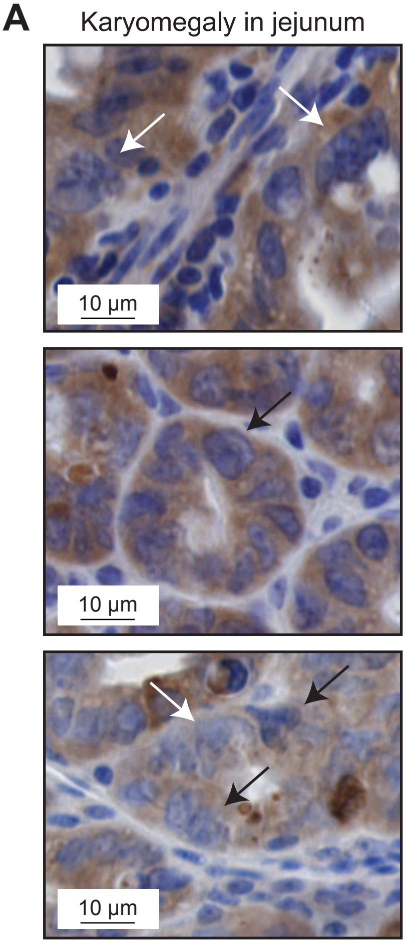

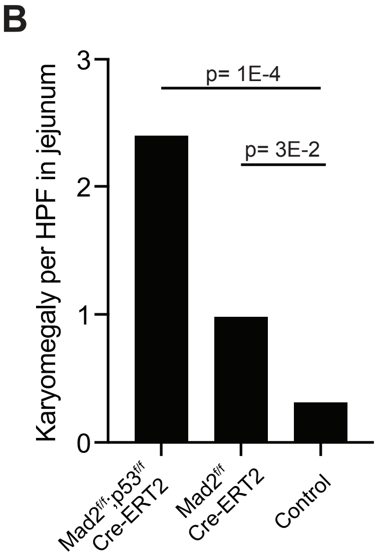

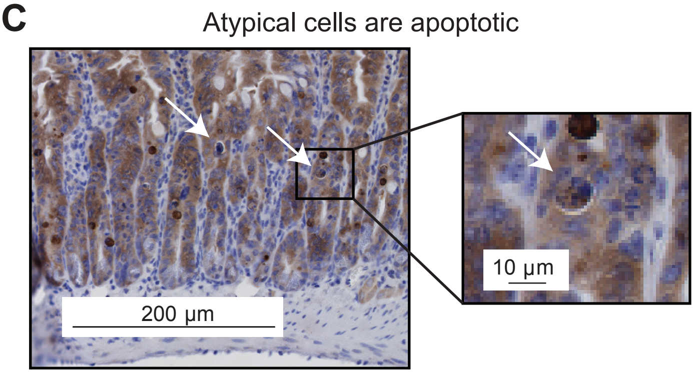

Schukken *et al*, Sup. Figure 2

Schukken *et al,* Supplementary Figure 3; multiple pages

Raw uncropped microscopy images and genomci PCR gel imaged used in the main and supplementary figures with reference to which figure the uncropped image contributed to.

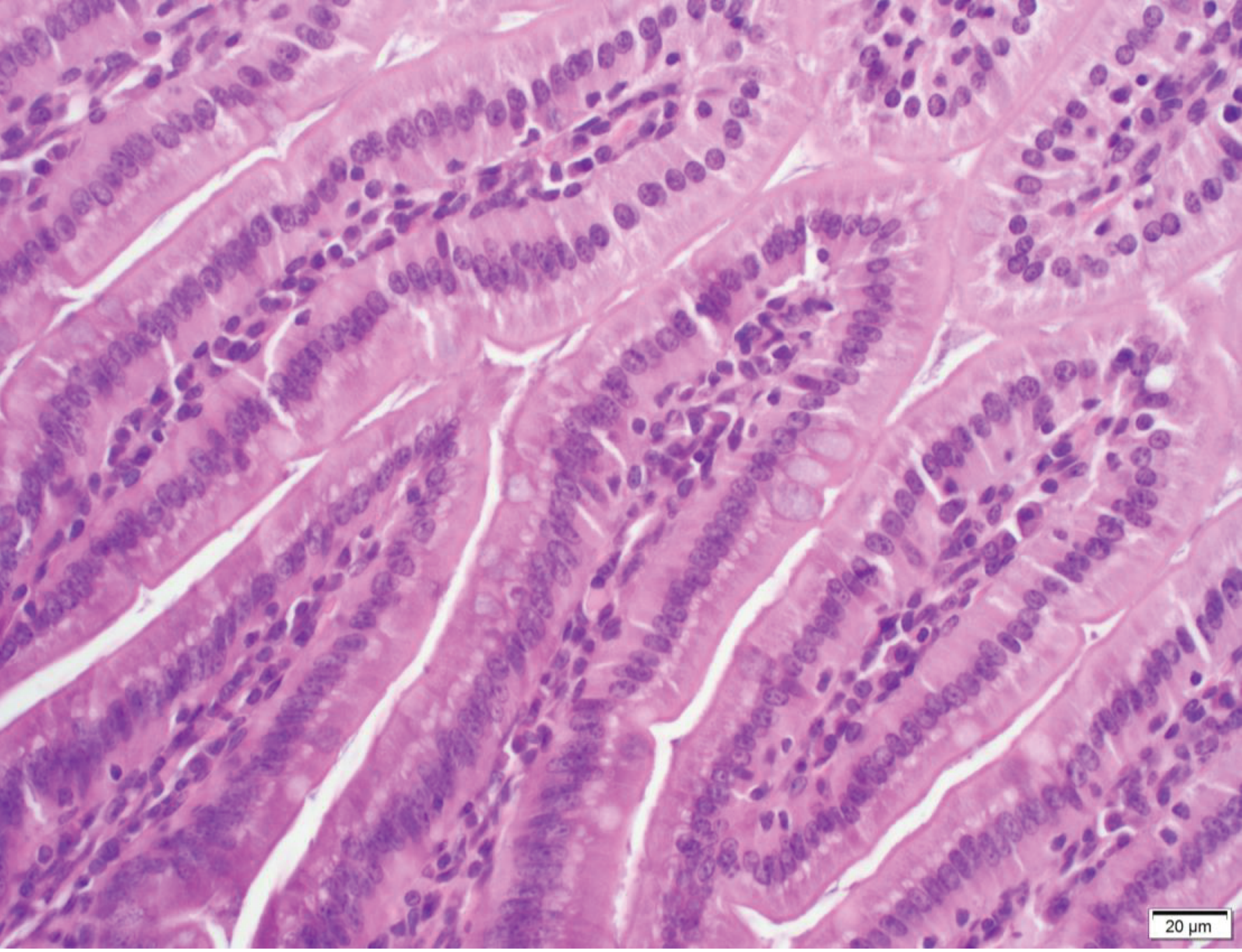

Figure 1 B, left

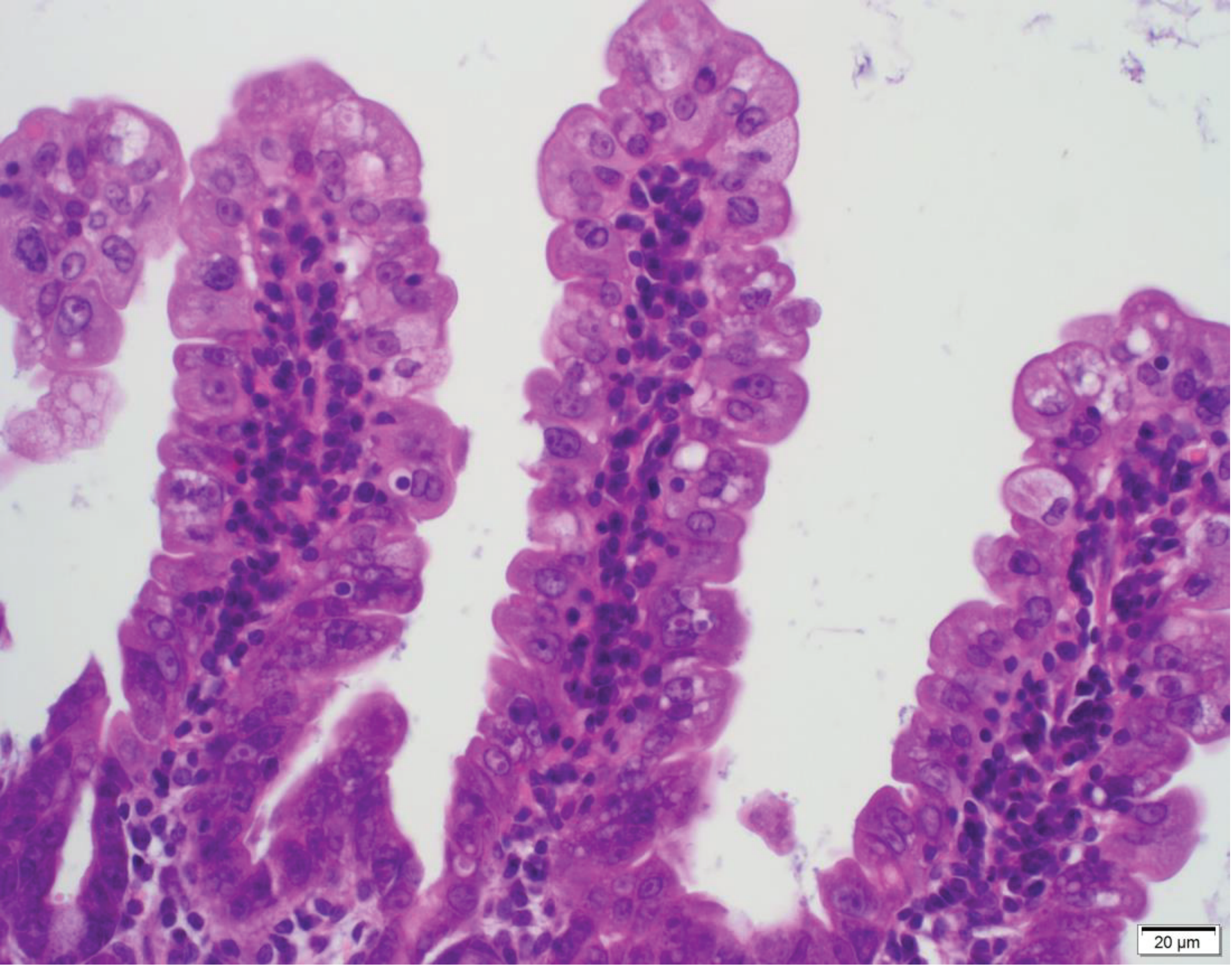

Figure 1 B, center

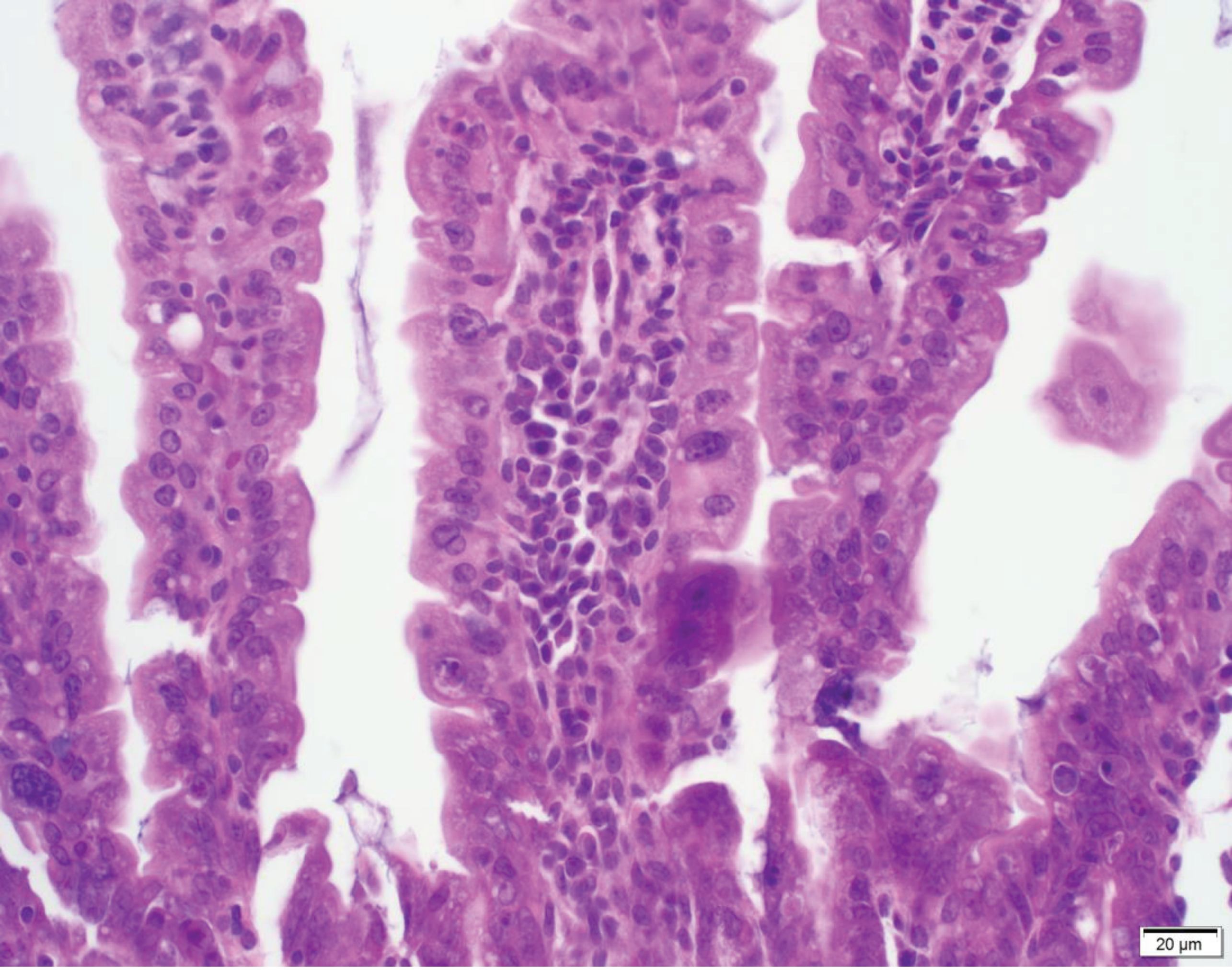

Figure 1 B, right

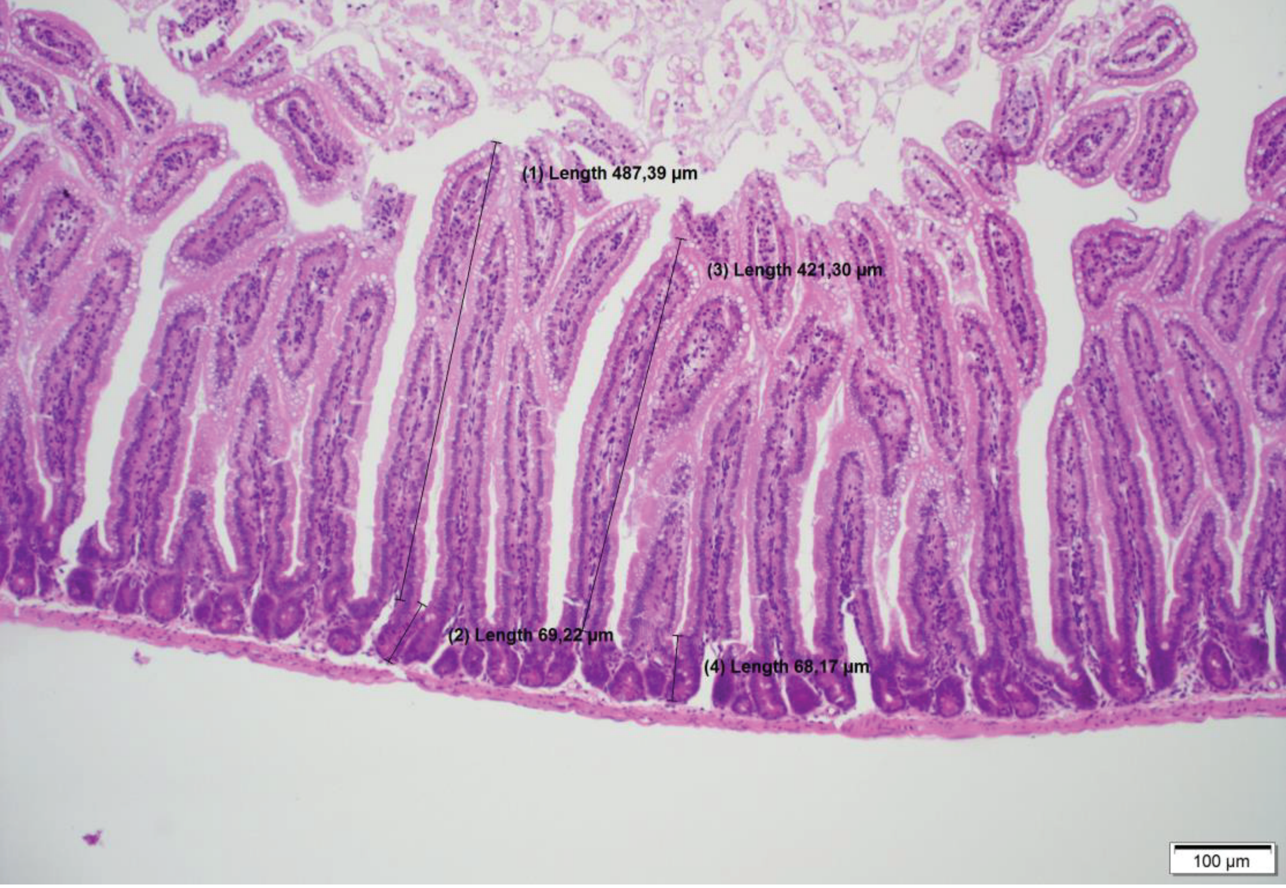
(1) Length 487,39 µm
(3) Length 421,30 µm
(2) Length 69,22 µm
(4) Length 68,17 µm
100 µm

Fig1 C, top

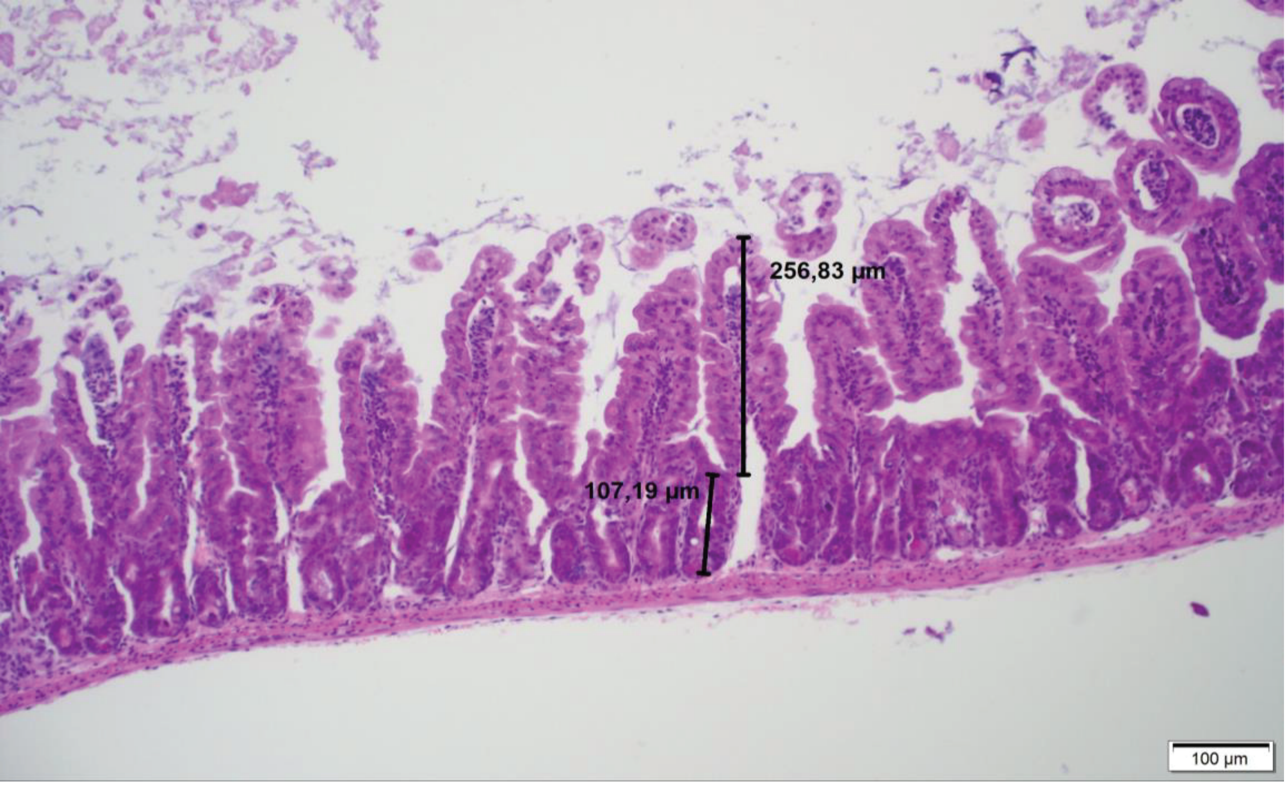

Figure 1 C, bottom

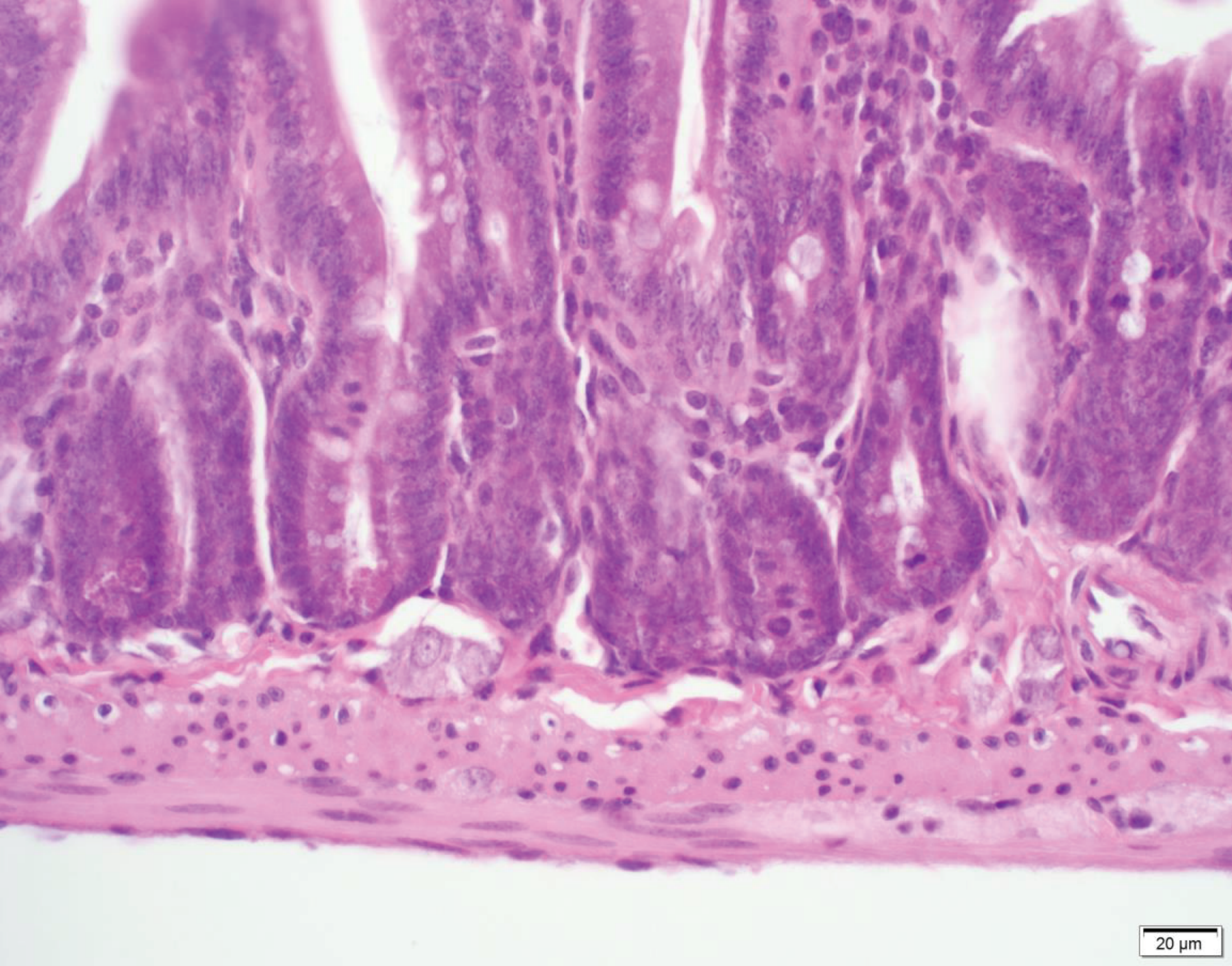

Figure 1 D, top

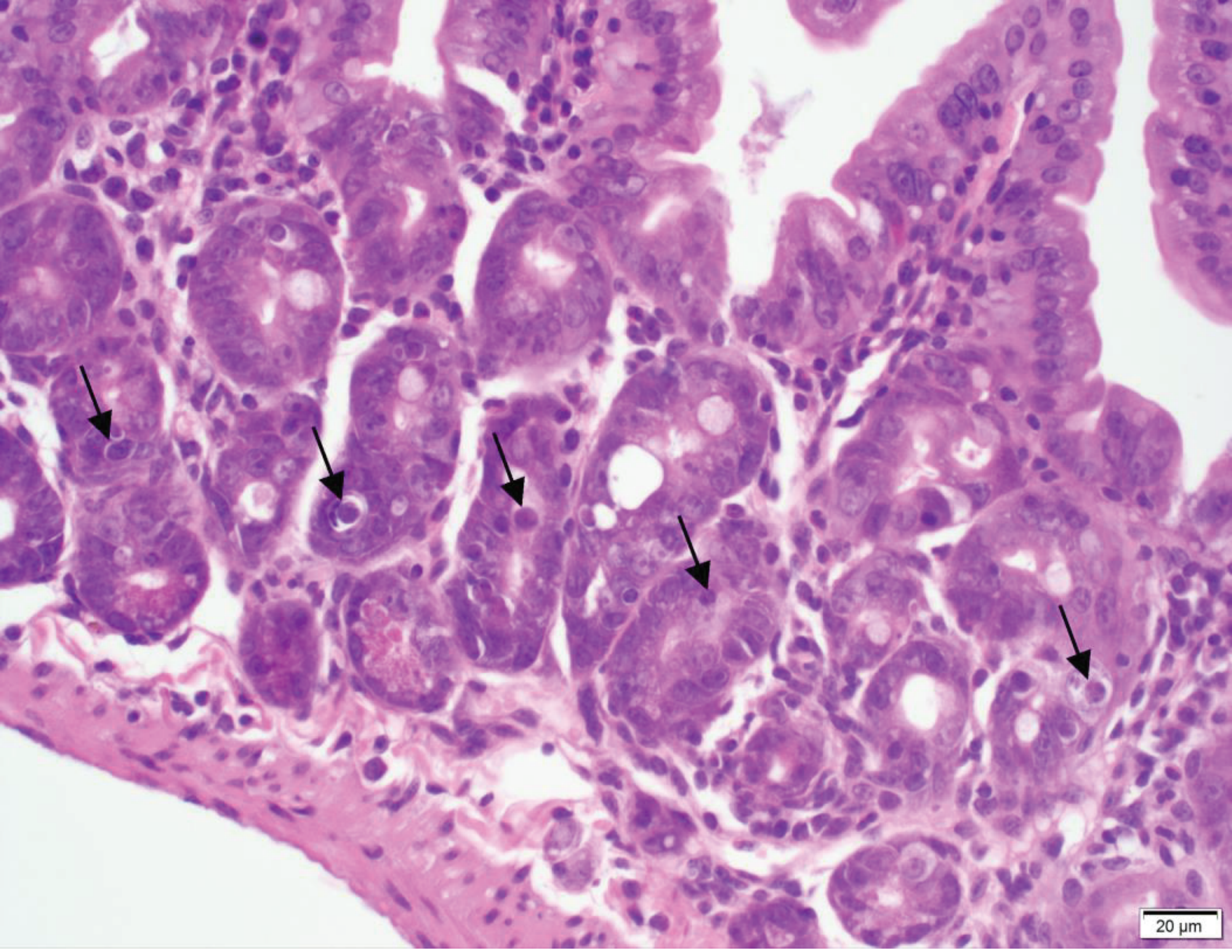

Figure 1 D, middle

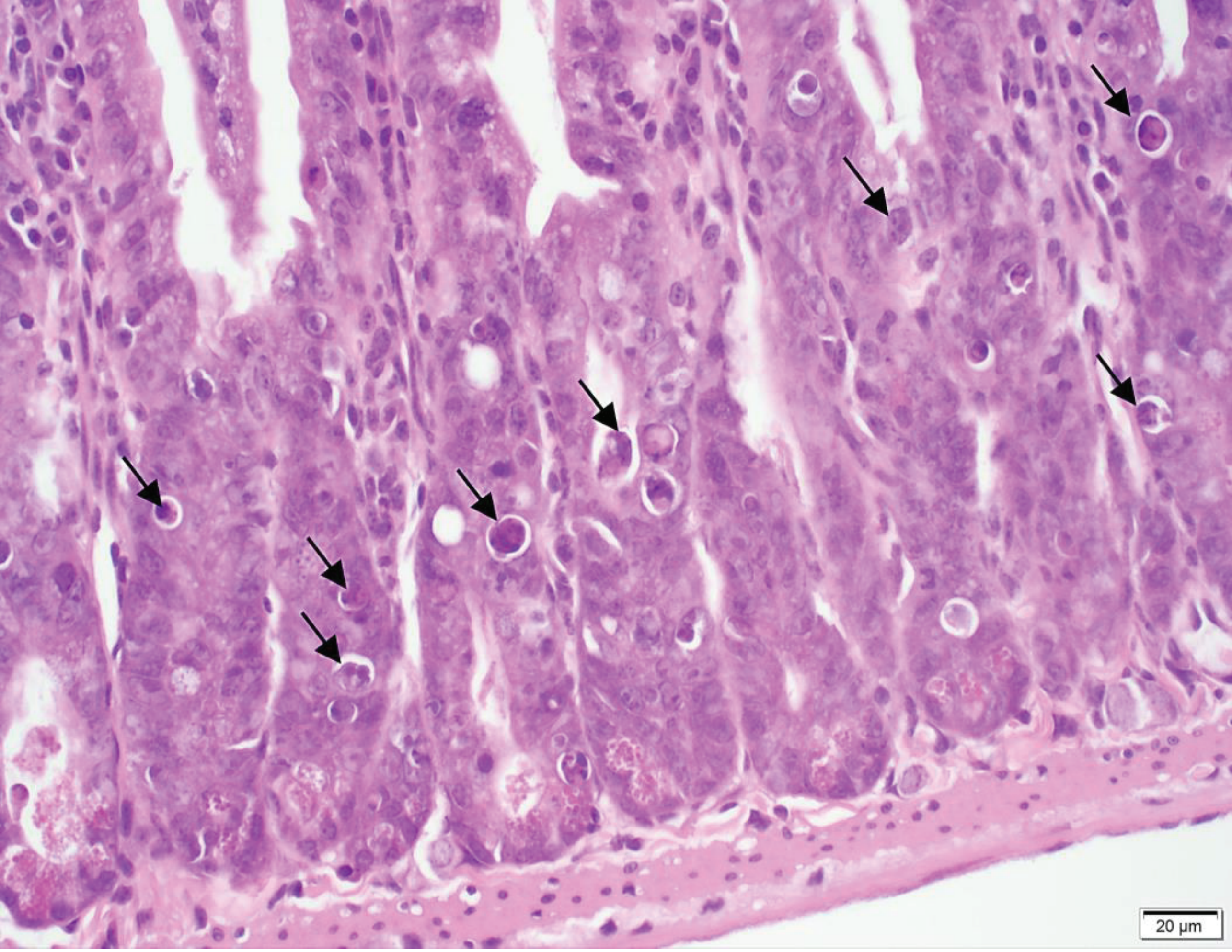

Figure 1 D, bottom

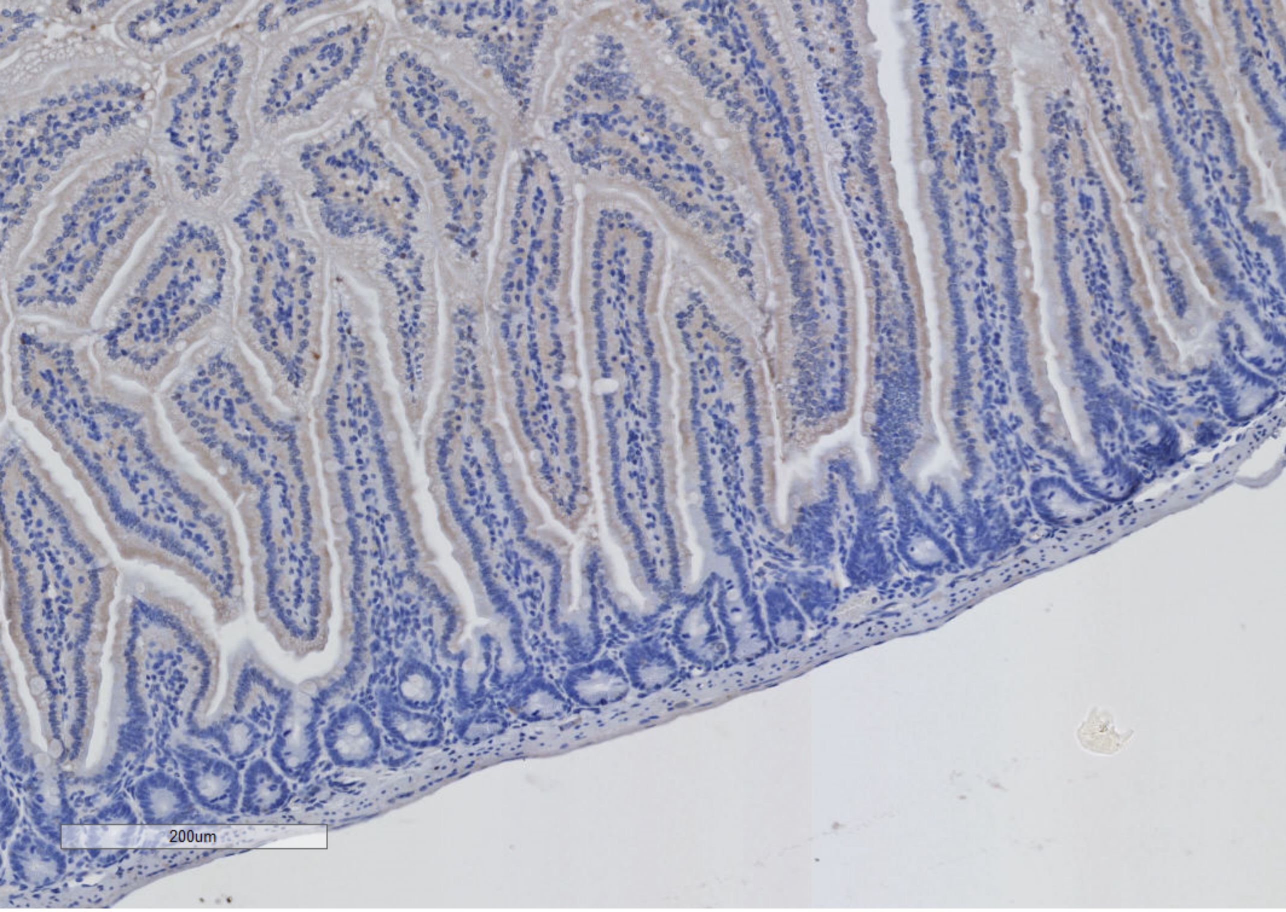

Figure 2 A, top

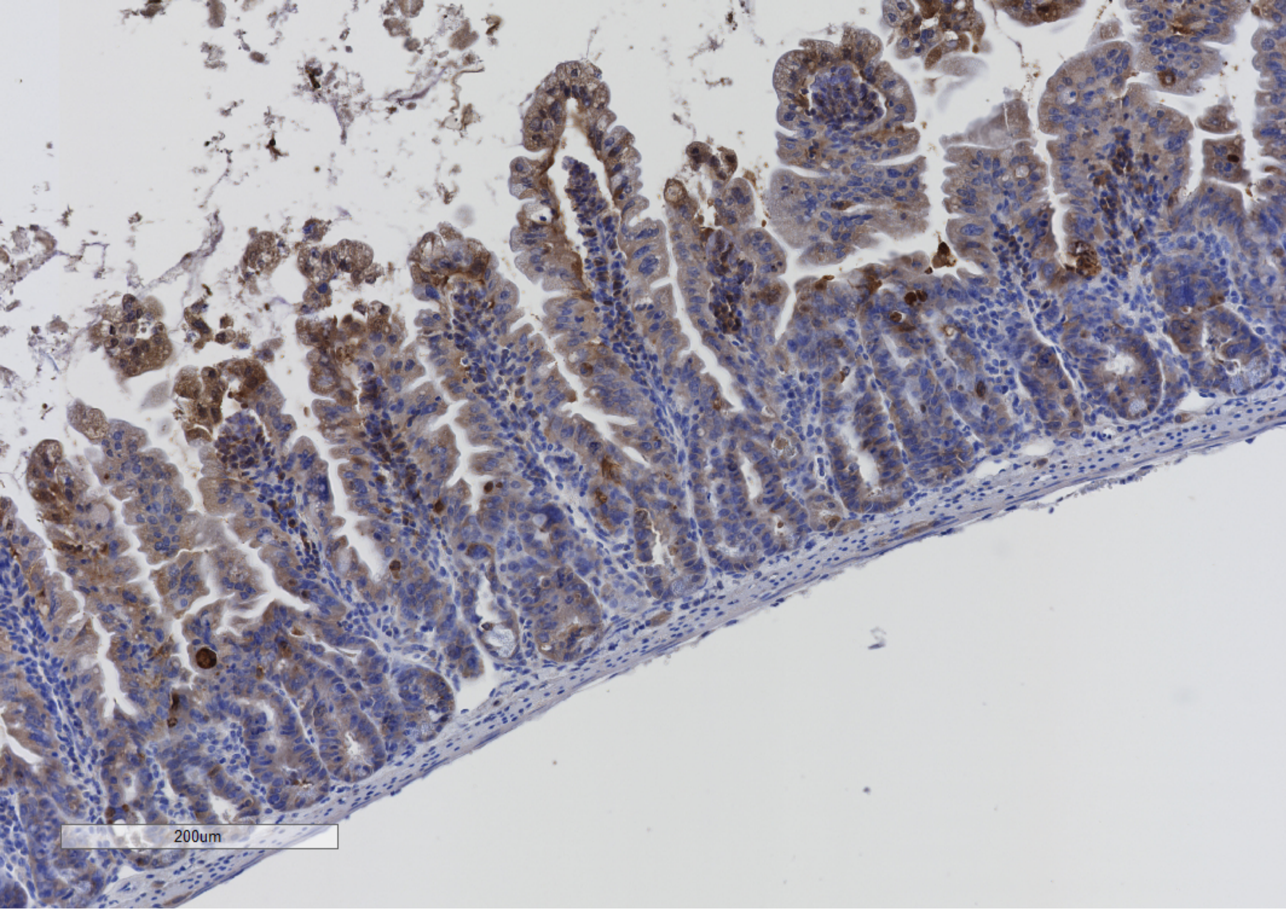

Figure 2 A, bottom

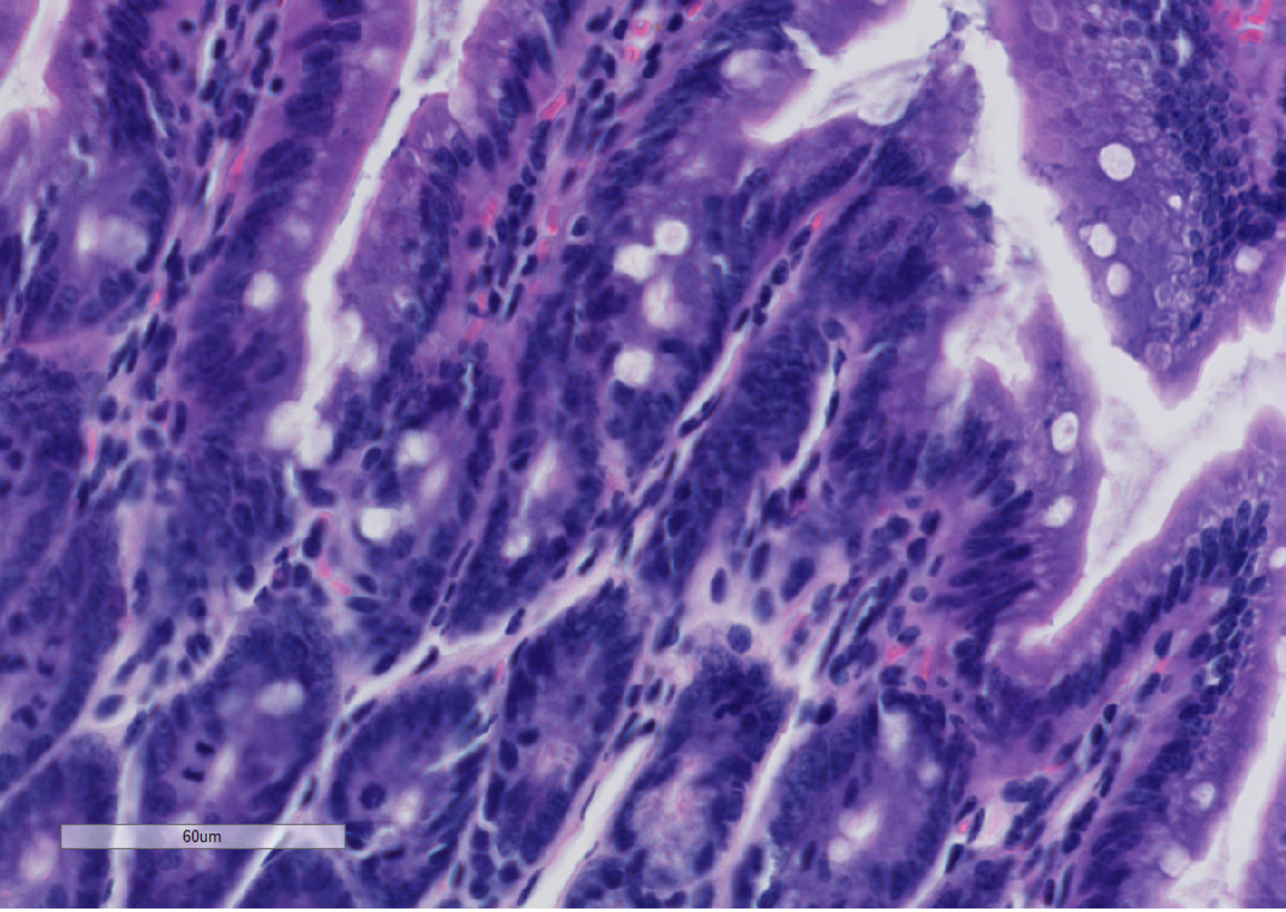

Figure 2 C, top

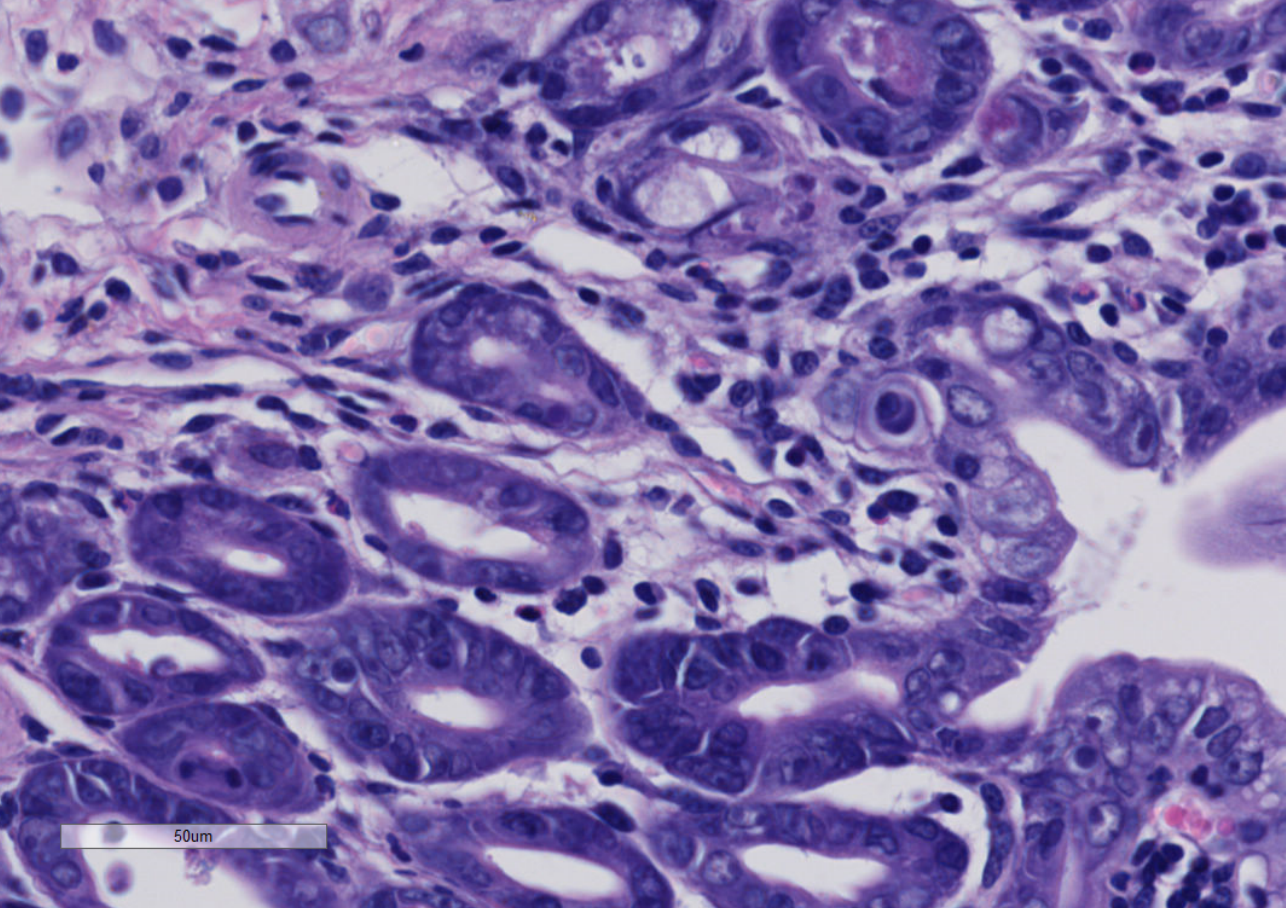

Figure 2 C, bottom

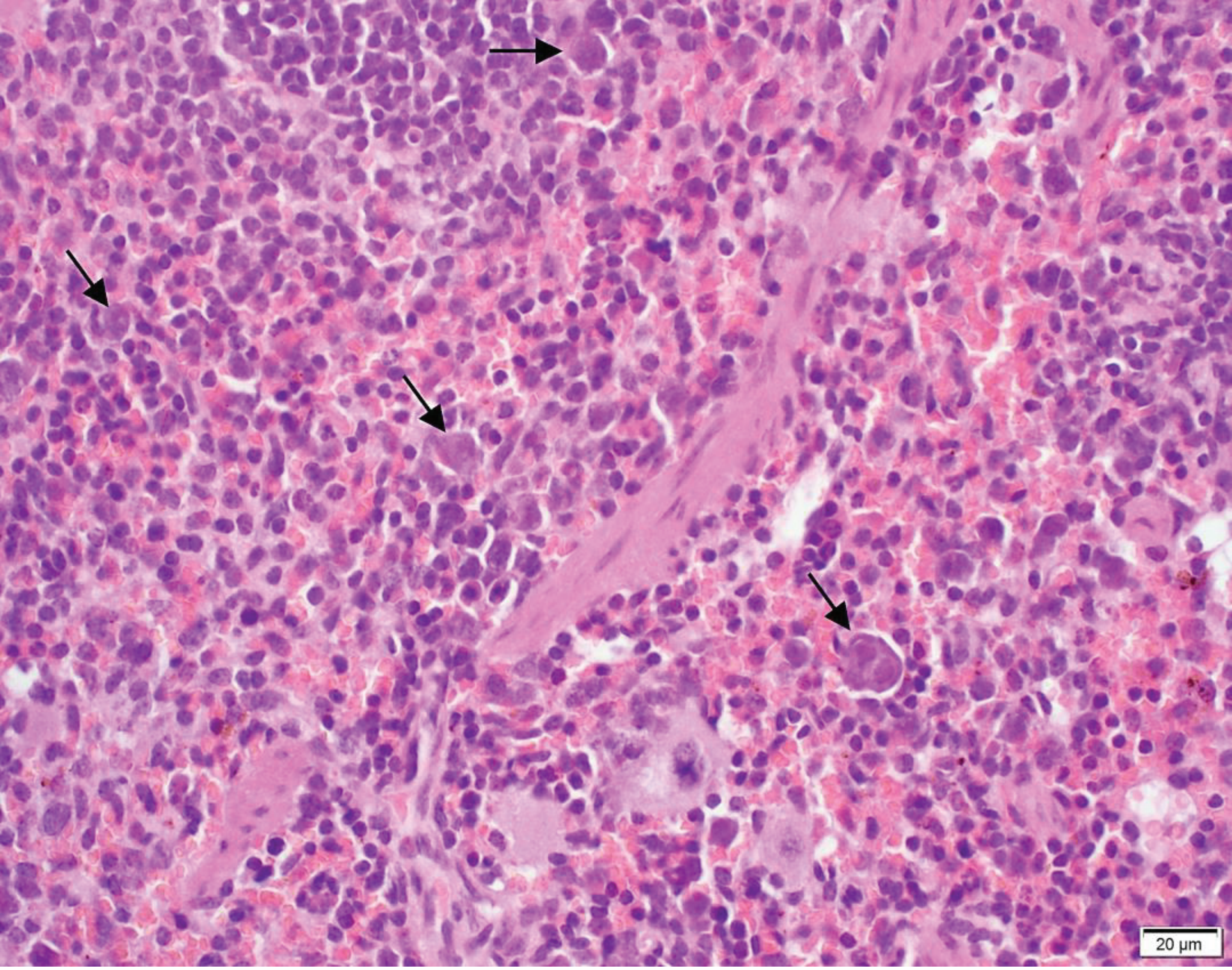

Figure 3 A

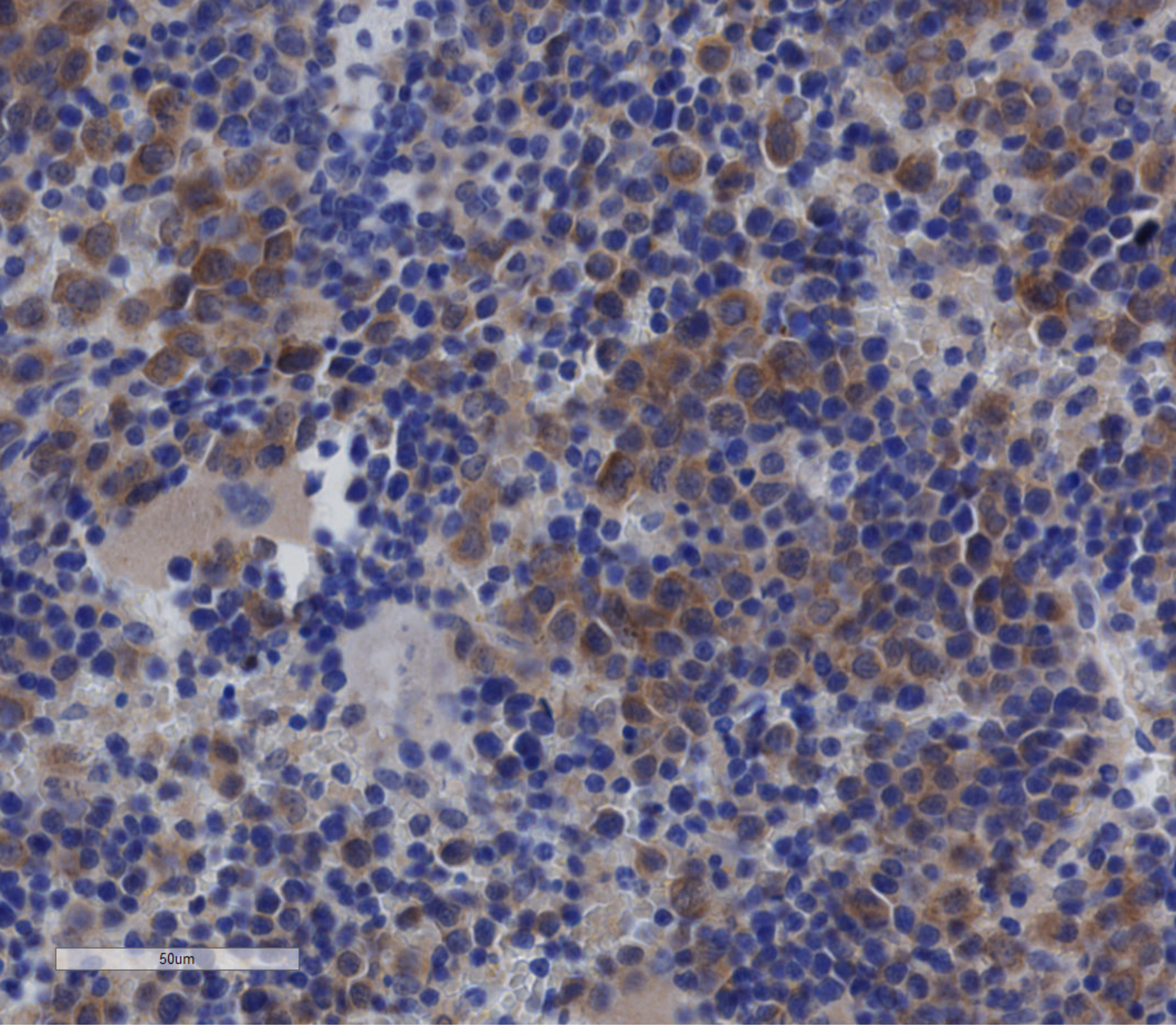

Figure 3 C, left

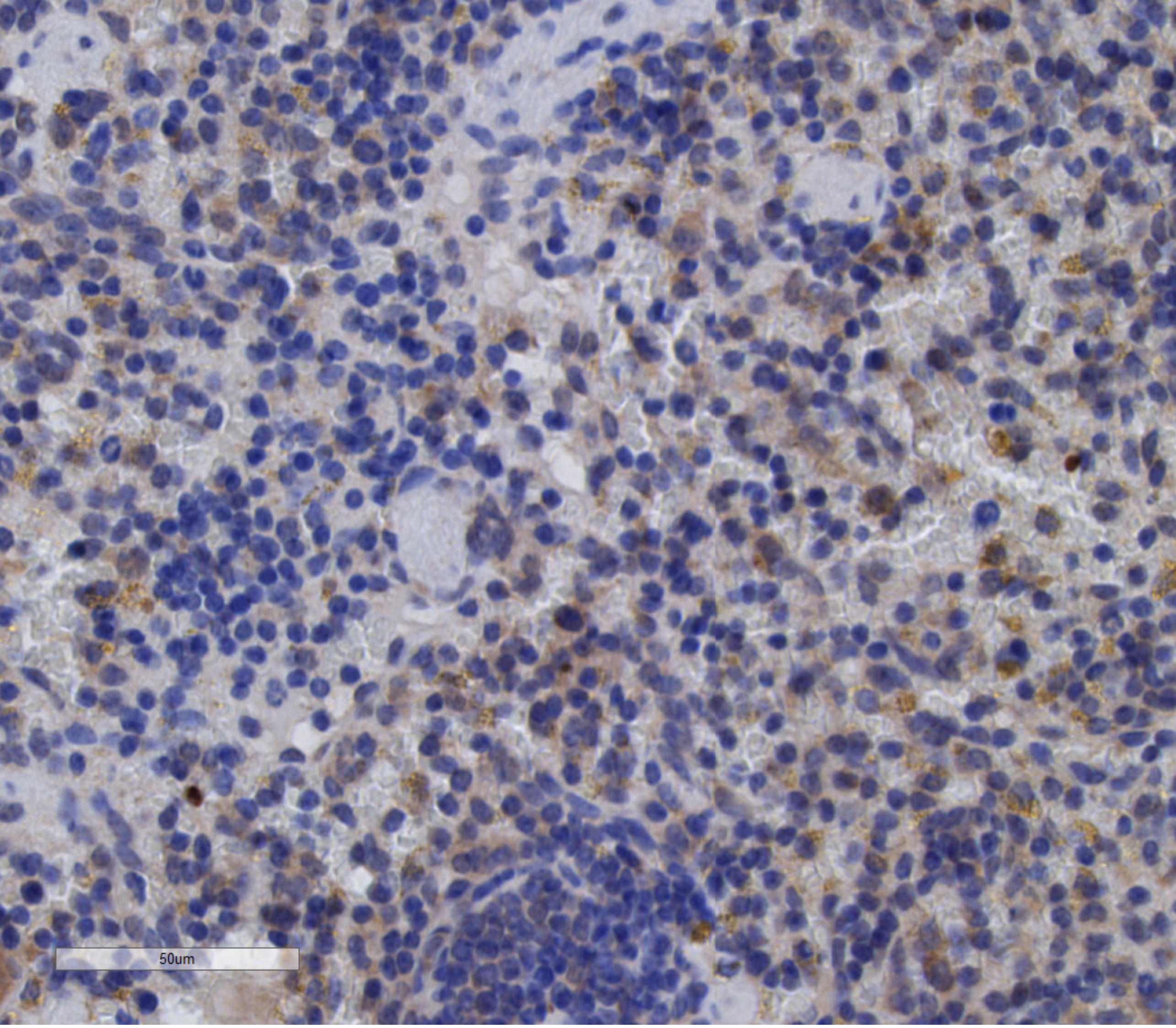

Figure 3 C, middle

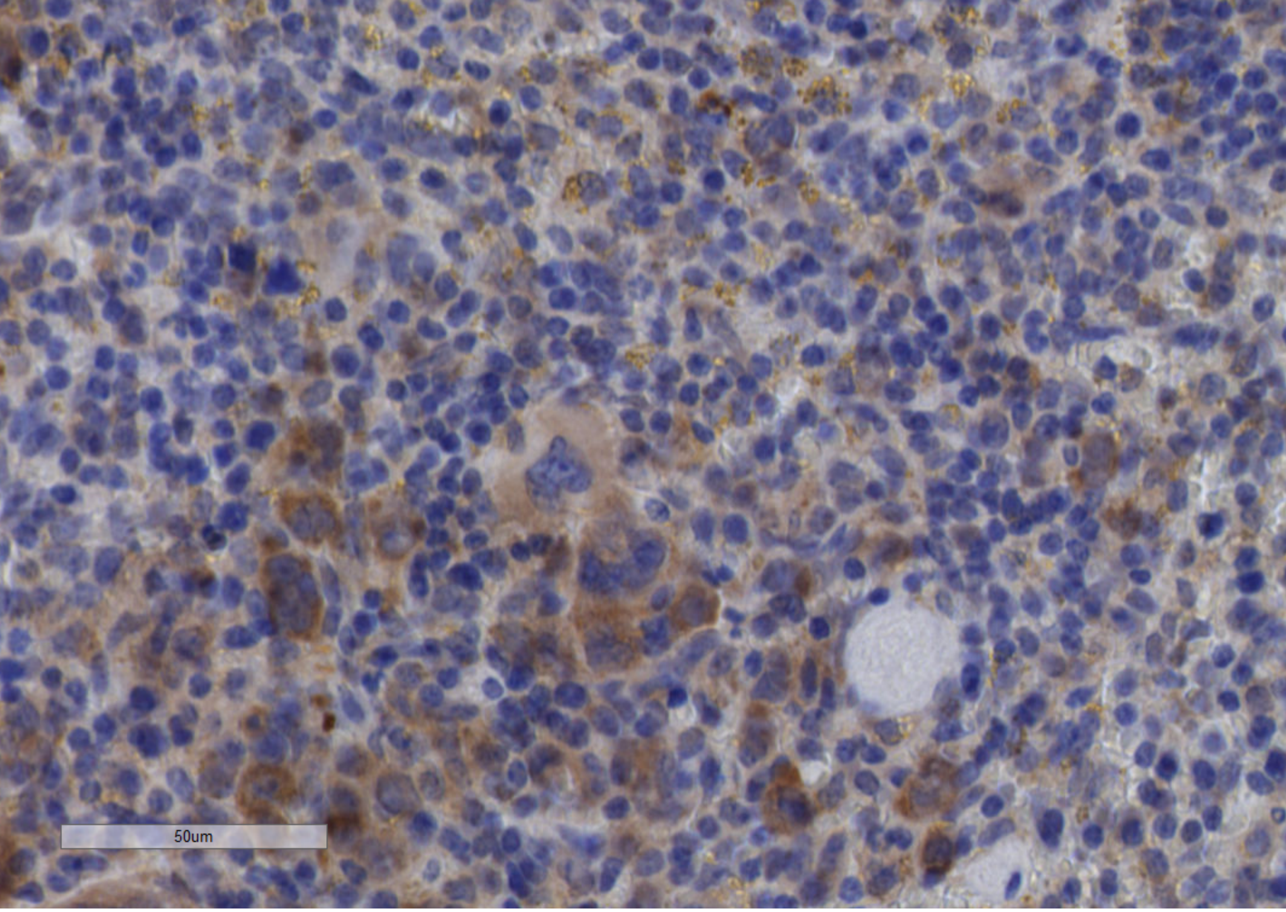

Figure 3, C, right

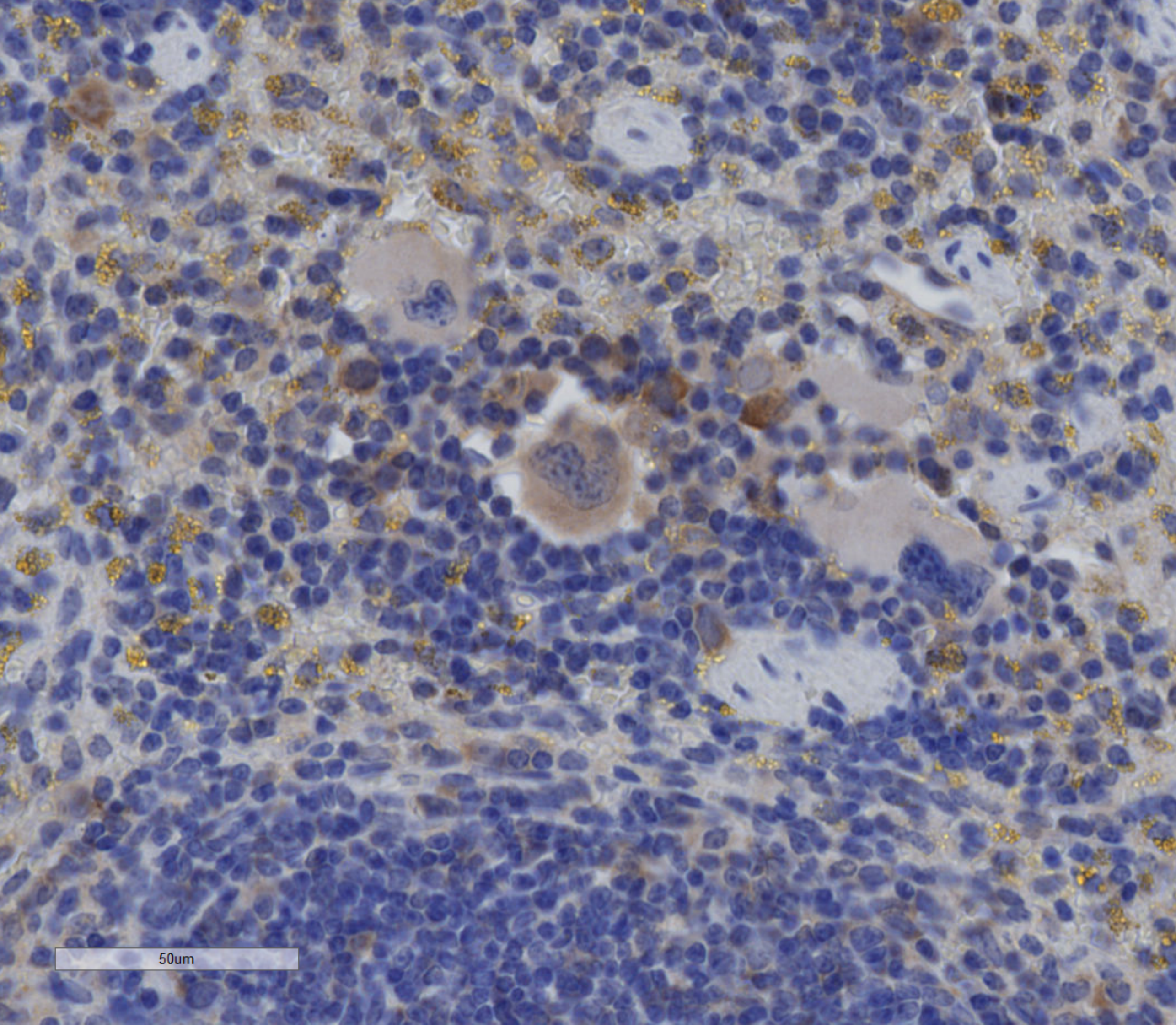

Figure 3 D

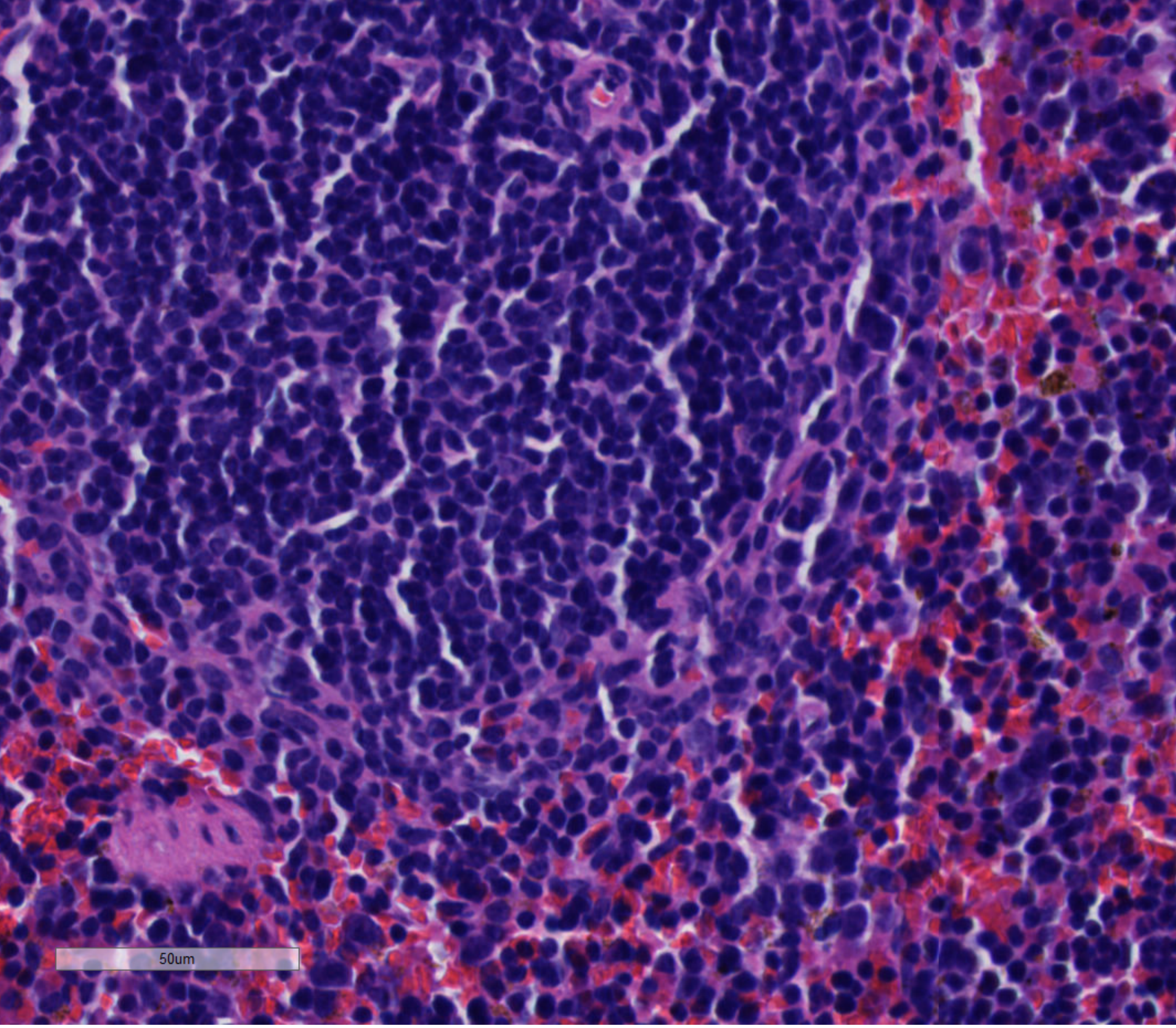

Figure 3 F, top

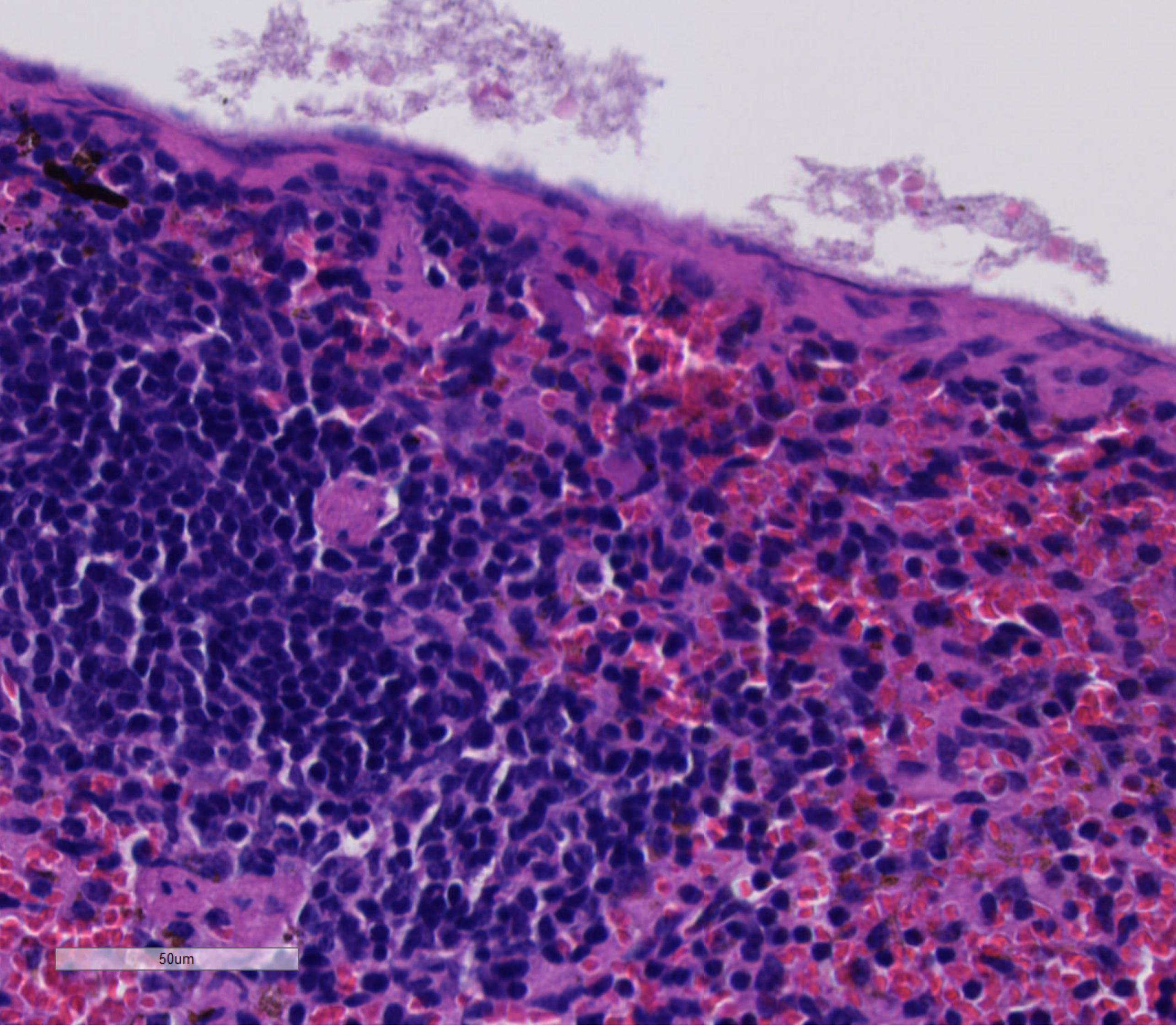

Figure 3 F, bottom

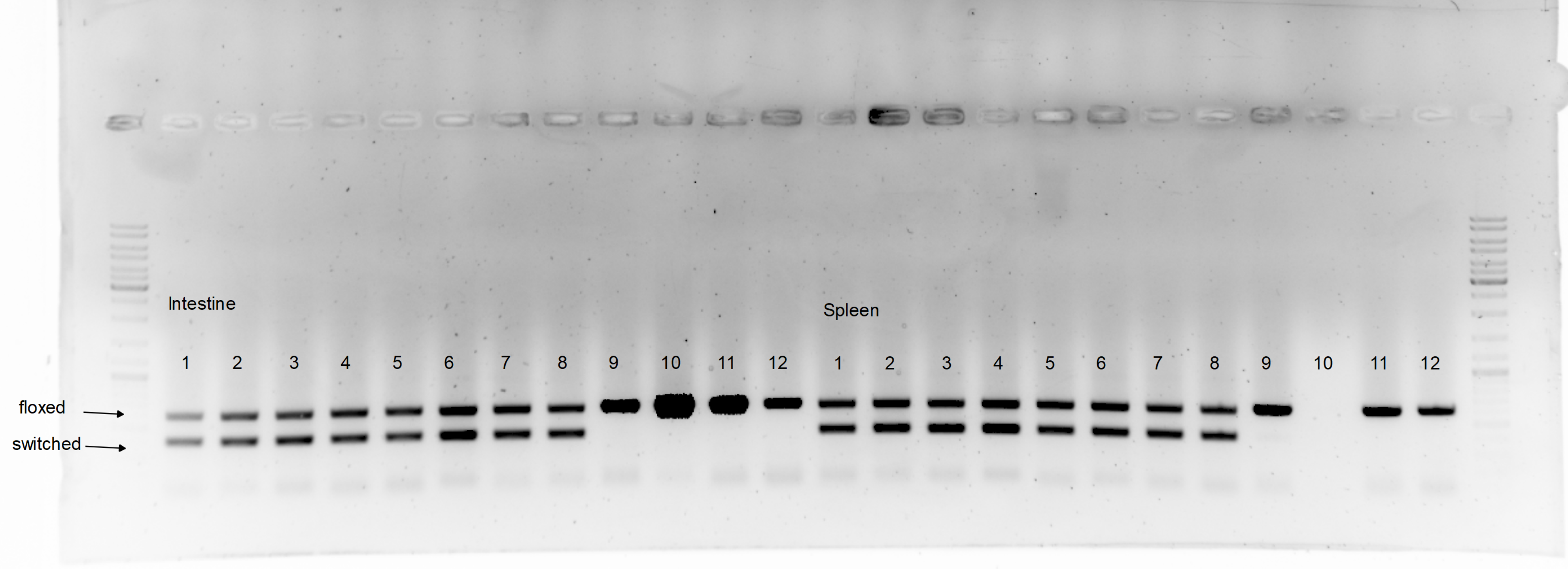

Supplementary Figure 1C

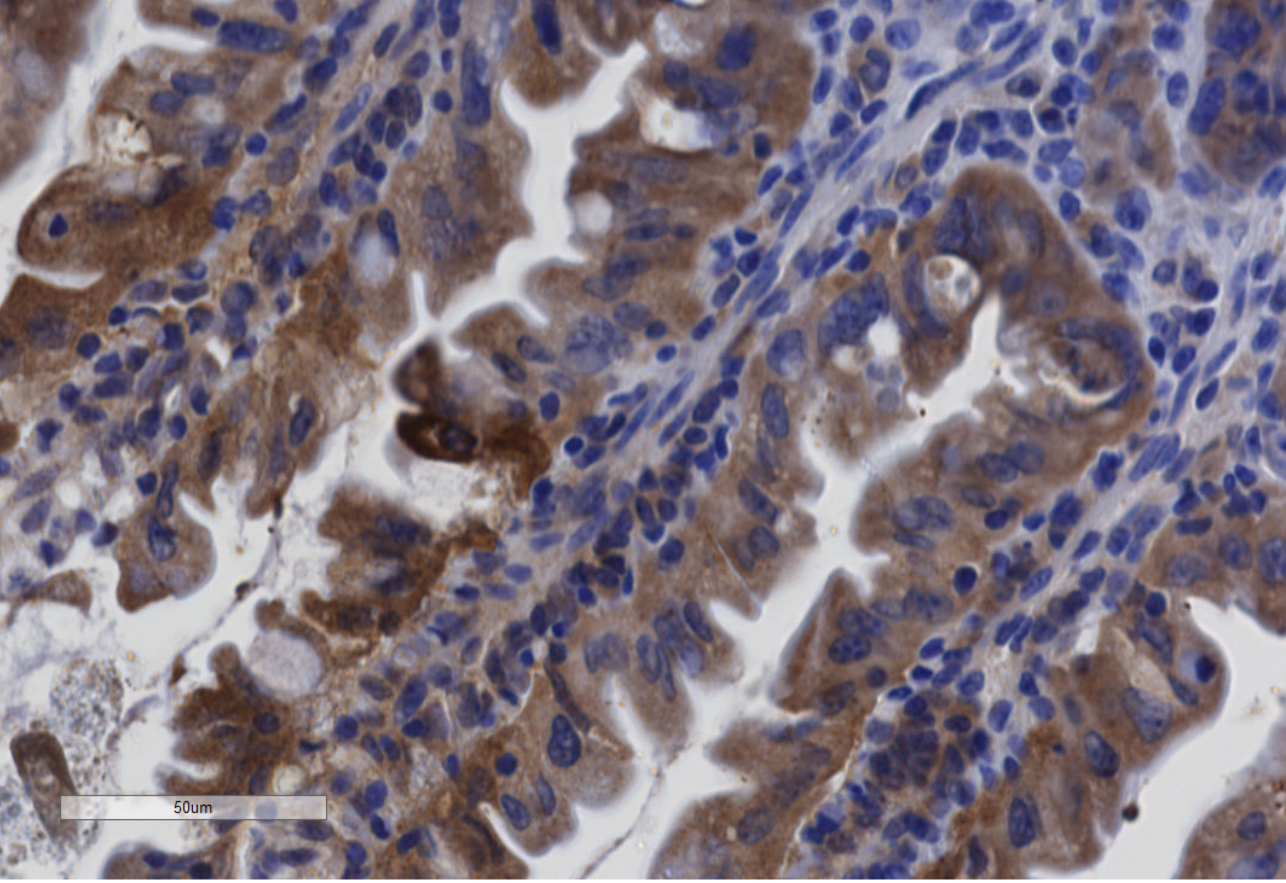

Supplementary Figure 2 A, top

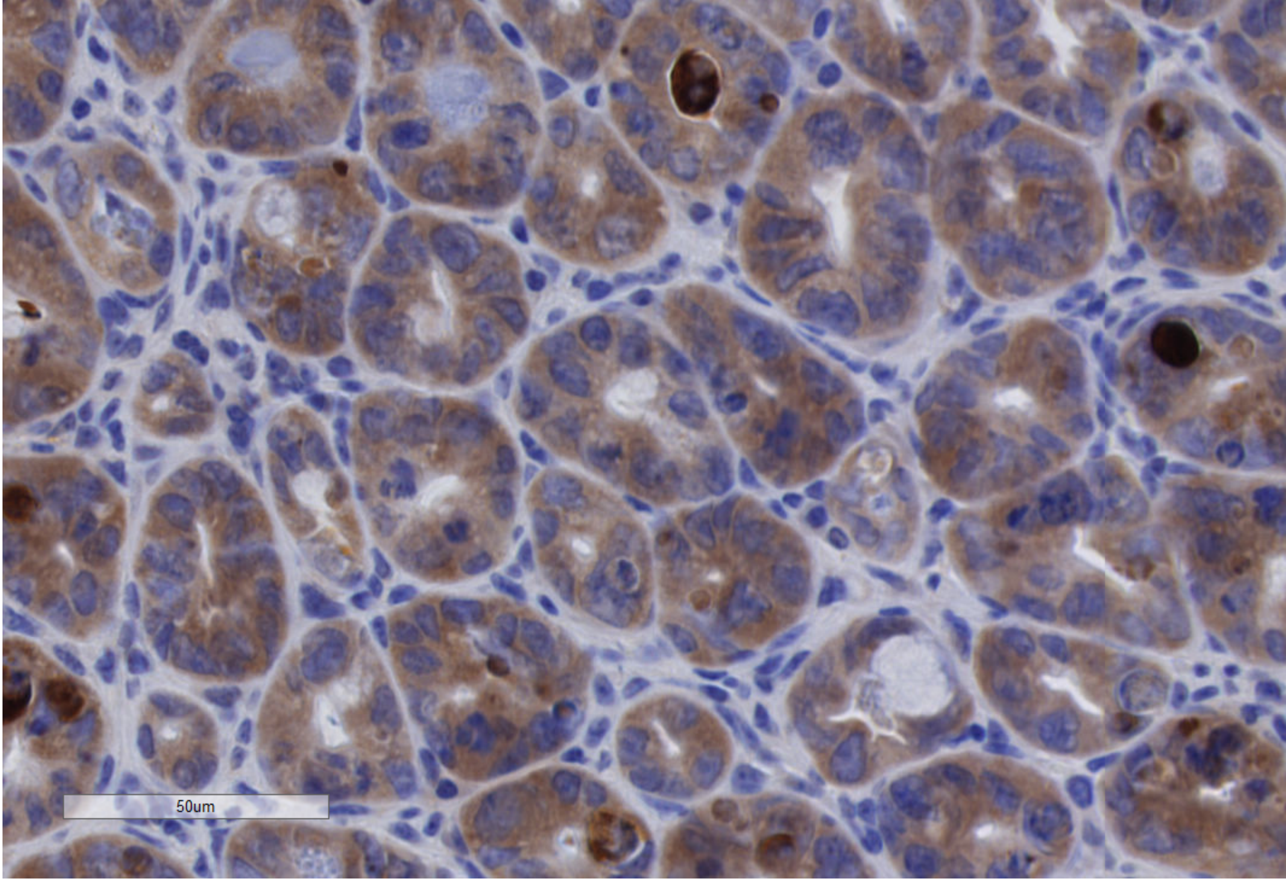

Supplementary Figure 2 A, middle

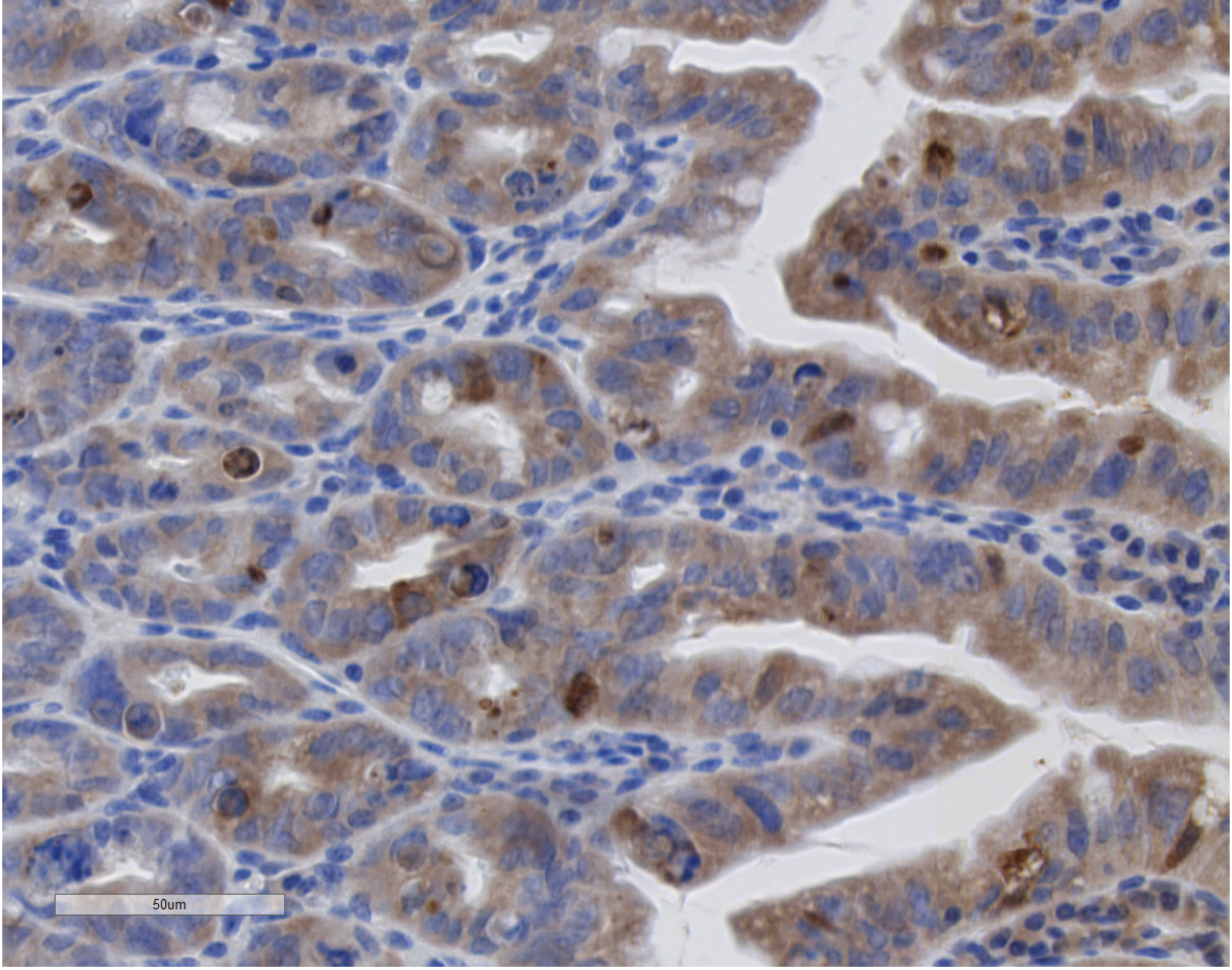

Supplementary Figure 2 A, bottom

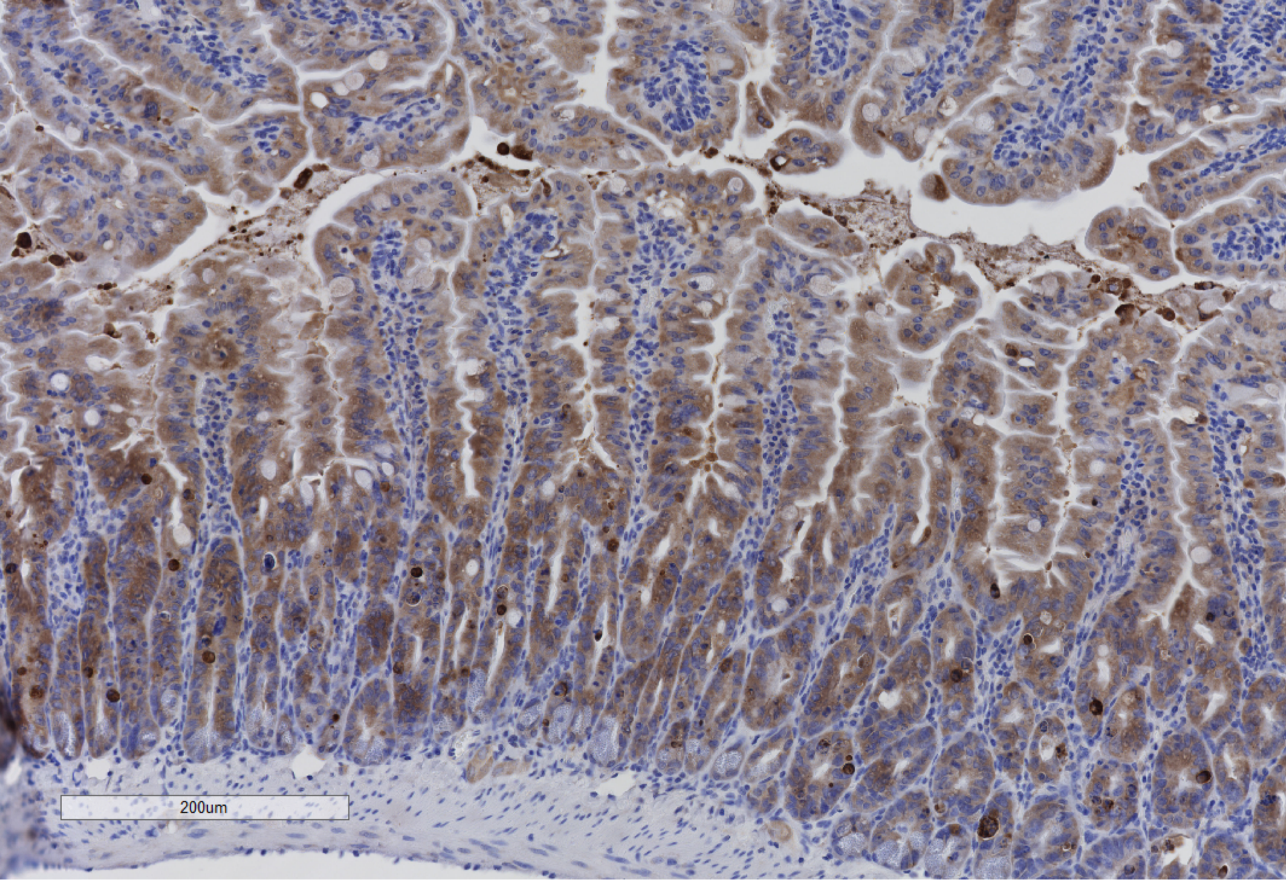

Supplementary Figure 2 C
